# Interannual Regime Shifts and Driver Thresholds of Terrestrial Ecosystem Vulnerability in Northwestern Sichuan of China Based on an XGBoost-SHAP Model

**DOI:** 10.3390/biology15040303

**Published:** 2026-02-09

**Authors:** Cuicui Jiao, Zonggui He, Juan Xu, Xiaobo Yi, Ji Luo, Ping Huang

**Affiliations:** 1 School of Economics, Sichuan University of Science & Engineering, Yibin 644000, China; 2 School of Economics, Guizhou University, Guiyang 550025, China; hezonggui1997@163.com; 3 College of Economics and Management, Hebei Agricultural University, Baoding 071000, China

**Keywords:** ecosystem vulnerability, interannual regime shifts, driver thresholds, XGBoost-SHAP model, northwestern Sichuan

## Abstract

TENS constitutes a critical ecological barrier on the southeastern margin of the Qinghai–Tibet Plateau, providing essential services such as water conservation and biodiversity protection and helping to safeguard water security in the upper reaches of the Yangtze and Yellow Rivers. Thus, elucidating its vulnerability dynamics is paramount for regional security. Integrating multi-source spatiotemporal data with an interpretable XGBoost–SHAP framework, we quantified interannual variation in vulnerability and the nonlinear threshold responses of key drivers. The results showed pronounced nonlinear phase changes in vulnerability, with 47.96% of the area experiencing abrupt shifts. Notably, 37.89% of TENS reversed from decreasing to increasing vulnerability. TENS underwent an intensive transition during 2010–2015. Interannual variability was dominated by the coupled influence of human disturbance, soil moisture, and atmospheric water, accounting for nearly 60% of the variation, and showed distinct thresholds. Grazing intensity < 0.90 SU/ha was a moderate disturbance, reducing vulnerability, but it became a stressor above this level. Soil moisture showed an inflection point at 79 mm, while vapor pressure deficit (VPD) < 0.39 kPa enhanced resilience, revising the view of VPD as solely a stress factor. Different ecosystems exhibited distinct driving mechanisms. Grasslands were controlled by shallow soil moisture and grazing, forests by hydrothermal balance, and wetlands by low-intensity anthropogenic disturbance (NTL as a proxy; e.g., tourism development or urban expansion). These findings highlight the risk of abrupt shifts in vulnerability regimes (turning points and trend reversals) and support management that emphasizes quality improvement and threshold-based risk management.

## Simple Summary

Northwestern Sichuan is a key ecological function zone in China, providing water conservation and biodiversity protection services and helping to safeguard water security in the upper reaches of the Yangtze and Yellow Rivers. Previous studies often oversimplified how ecosystems change, missing sudden shifts in their health. This study used advanced computer modeling to analyze the vulnerability of these terrestrial ecosystems to damage. We found that nature does not change in a straight line, while nearly half the region experienced abrupt shifts. Alarmingly, more than 37% of the previously restored territory has begun to deteriorate again since 2010. This suggests that early restoration efforts, like planting trees, may have reached their limits. We discovered specific “tipping points” where factors like grazing and soil moisture change from being helpful to harmful. For example, while moderate grazing supports grasslands, excessive grazing damages them. These findings are vital because they show that simply expanding vegetation quantity is not enough. To protect these diverse landscapes, society must shift focus to improving ecological quality and strictly managing these risk thresholds. This approach will better safeguard regional biodiversity and water security for the future.

## 1. Introduction

Climate change has emerged as one of the primary challenges confronting global biodiversity, with particularly pronounced impacts on terrestrial ecosystems [1,2]. Vulnerability, as a key metric for assessing an ecosystem’s self-regulatory capacity in response to external disturbances, has become a critical topic in ecology and global change research [3]. Northwestern Sichuan, situated at the transitional zone between the southeastern margin of the Qinghai–Tibet Plateau and the Sichuan Basin, exhibits complex topography and diverse habitat types, serving as a biodiversity hotspot and a pivotal region for ecosystem services. Meanwhile, this area is susceptible to climate change, characterized by fragile habitats and complex human-environment interactions. Therefore, a comprehensive analysis of the driving factors underlying the spatiotemporal characteristics of vulnerability in northwestern Sichuan’s terrestrial ecosystems under climate changes holds significant scientific and practical value. The present study aimed to elucidate the regional ecological response mechanisms, evaluate the ecological risks, and formulate adaptive strategies designed to lessen the vulnerability of the various habitats.

Extensive research has been conducted on the vulnerability of terrestrial ecosystems. This research has evolved from early global macro-assessments [4] to regional empirical analyses [5], and from static evaluations to explorations of dynamic mechanisms. The theoretical foundation of this field centers on the three-dimensional framework of exposure–sensitivity–adaptive capacity (resilience). Further, approaches, such as remote sensing techniques [6] and probabilistic models [7], have been used to quantify the spatiotemporal patterns and driving factors of vulnerability. Gonzalez et al. [4] integrated long-term climate observations with a dynamic global vegetation model to investigate the vulnerability of terrestrial ecosystems to biome shifts under different greenhouse gas emission scenarios. They revealed widespread vulnerability to changes in global ecosystems, highlighting the impacts of shifts in biome distributions. Subsequent research incorporated the Copula probability framework and climate variability calculations into regional-scale vulnerability assessments [7]. Fang et al. [7] proposed a bivariate probability framework based on Copulas, enabling a systematic evaluation of the spatiotemporal vulnerability of vegetation on the Loess Plateau to drought stress. This approach facilitated a shift from deterministic correlation analysis to conditional probability risk assessment [7]. Since 2020, vulnerability studies have increasingly incorporated dynamic changes [8,9], human activity factors such as land use changes [10,11,12], and lagged effects of climate change [13]. Yin et al. [13] quantified the impact of lagged effects on the vulnerability of vegetation to drought at a global scale using the Standardized Precipitation Evapotranspiration Index and Normalized Difference Vegetation Index (NDVI). Their results indicated that 68.22% of terrestrial vegetation exhibited a 1–3 month lagged response to drought, and neglecting lagged effects leads to a general underestimation of vegetation vulnerability [13]. Zhang et al. [9] examined the spatiotemporal dynamics of ecological vulnerability in the Yellow River Basin and its response mechanisms to land–cover changes using the exposure–sensitivity–resilience framework. Their study showed that the basin displayed a trend toward ecological degradation, and projects such as the Grain for Green and Grazing to Grassland programs improved the environment. Still, it did not reverse the overall degradation [9]. Zhang et al. [8] analyzed ecological vulnerability on the Qinghai–Tibet Plateau under the exposure–sensitivity–resilience framework. The results demonstrated that the eastern and central regions were high-vulnerability areas; the western region showed spatial heterogeneity, and precipitation exerted a significantly greater influence on vulnerability than temperature [8].

Despite considerable advancements in this field, existing studies still have limitations in three key aspects: (1) in the construction of a theoretical framework, most studies rely on static assessment models and fail to incorporate non–linear feedbacks; (2) regarding research methods, the majority of studies depend on traditional analytical approaches such as linear regression or geographical detectors, lacking the integration of innovative techniques such as machine learning to reveal the non-linear relationships between vulnerability and influencing factors or to identify response thresholds; and (3) in terms of research scale and object selection, prior work has primarily focused on global, national, or typical regions such as the Qinghai–Tibet Plateau and the Loess Plateau, while systematic investigations of plateau-mountain transition zones, such as in northwestern Sichuan in China, remain scarce.

Northwestern Sichuan lies on the southeastern margin of the Qinghai–Tibet Plateau and serves as a transition zone. All 31 counties (cities) in this area are designated national key ecological function zones. The region provides essential services such as water conservation, biodiversity protection, and the maintenance of the ecological barrier along the upper reaches of the Yangtze and Yellow Rivers. Because its ecosystem vulnerability directly affects national water security and carbon sink capacity, a detailed assessment of terrestrial ecosystem vulnerability in northwestern Sichuan is essential to support China’s dual-carbon targets and the construction of an ecological civilization.

In the present study, we propose the following hypothesis. Interannual variation in terrestrial ecosystem vulnerability in northwestern Sichuan does not exhibit a simple linear or monotonic trend across all areas under the joint influences of climate change and human activities. Instead, nonlinear dynamics occur in some regions. The vulnerability response to climatic and anthropogenic drivers is likewise not a linear function, but rather it reflects a complex mechanism with apparent threshold effects. To test our hypothesis, this study systematically evaluated terrestrial ecosystem vulnerability in northwestern Sichuan under climate change by integrating multiple data sources, including vegetation indices, climate variables, and indicators of human activity. We employed machine learning methods as the primary analytical framework. The study had three main objectives: (1) to characterize the temporal dynamics of vulnerability, identifying long-term trends and potential abrupt shifts; (2) to quantify the relative contributions of climate change and human activities to interannual variation in vulnerability and assesses their threshold effects, and (3) to compare interannual vulnerability among various habitat types and determine the dominant driving factors, thereby elucidating the mechanisms underlying the formation of vulnerability and potential risk thresholds. The findings will provide a basis for regional ecological management and the design of climate adaptation strategies.

## 2. Materials and Methods

### 2.1. Study Area

**Figure 1:**
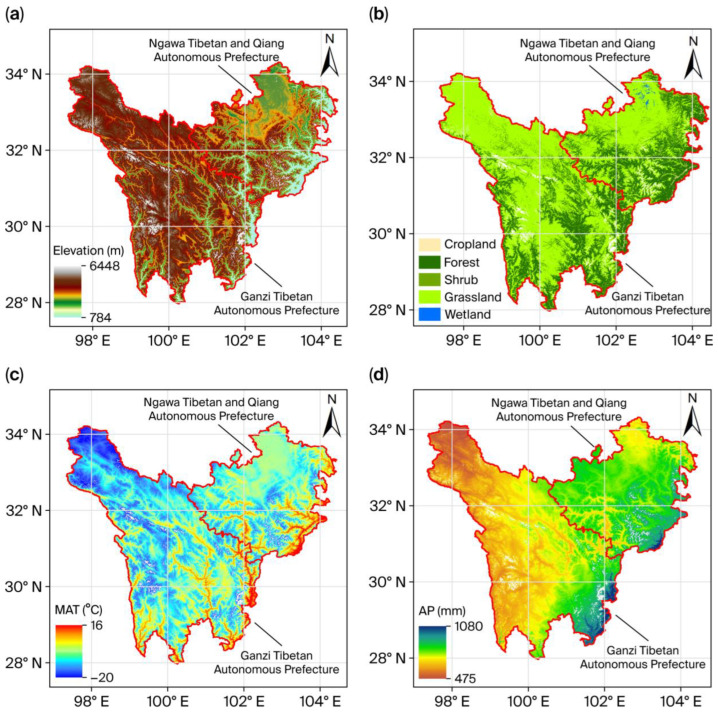
General geographic information of the TENS region. (**a**) Spatial distribution of the elevation; (**b**) Spatial distribution of various ecosystems; (**c**) Spatial distribution of mean annual temperature (MAT); and (**d**) Spatial distribution of annual precipitation (AP). The white areas in the figure represent No data regions, which include non-vegetation regions such as water bodies, snow and ice, bare land, and impervious surfaces. Figure 1 was derived from the study by Jiao et al. [14].

The Terrestrial Ecosystem of Northwestern Sichuan (TENS) comprises the Ganzi Tibetan Autonomous Prefecture and the Ngawa Tibetan and Qiang Autonomous Prefecture [14], situated in the Hengduan Mountains on the eastern edge of the Tibetan Plateau (27°56′–34°20′ N, 97°21′–104°27′ E). The landscape, which covers approximately 236,000 km^2^, slopes from northwest to southeast across a complex terrain and ranges in elevation from 3500 to 4500 m (Figure 1a). Five principal ecosystems occur in the region, namely, cropland, forest, grassland, shrubland, and wetland. Grassland is the most extensive, accounting for 63.82% of TENS, while forest covers 31.76% (Figure 1b). Major vegetation types include coniferous forest, broadleaf forest, mixed coniferous–broadleaf forest, alpine meadow, and alpine steppe. The regional climate features long, cold winters and lacks a distinct hot summer, instead showing generally cool, temperate conditions. Mean annual temperatures span from −22 °C to 16 °C (Figure 1c), and annual precipitation ranges from 475 mm to 1080 mm (Figure 1d).

### 2.2. Methods

**Figure 2:**
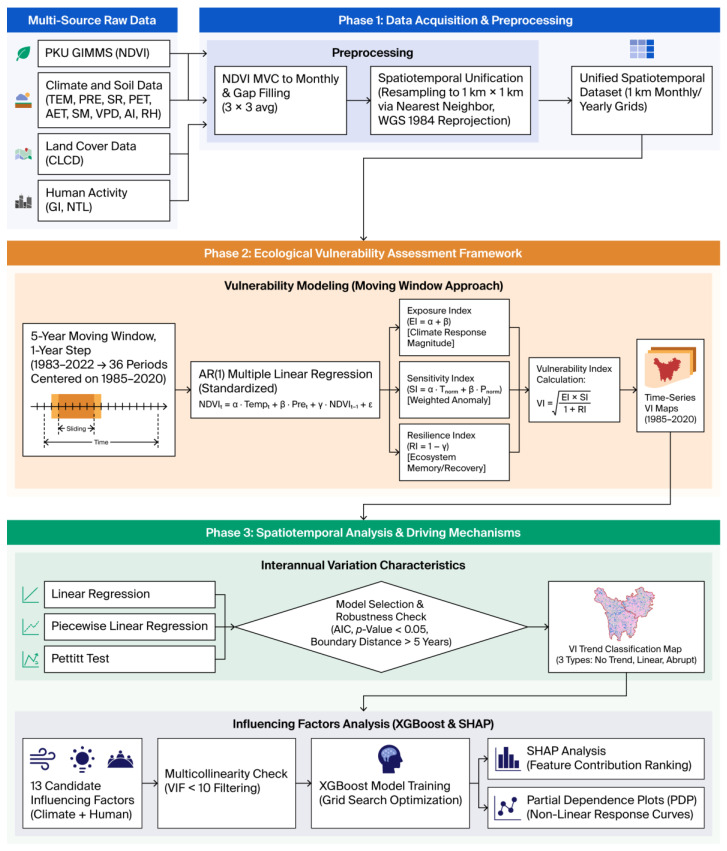
Technical flowchart for the study methods.

Figure 2 presents the technical flowchart outlining this study’s research framework, including the required data, preprocessing procedures, vulnerability assessment methods, and analytical approaches used to examine the temporal dynamics of vulnerability and their driving factors. Detailed descriptions of these methods appear in Section 2.2.1, Section 2.2.2, Section 2.2.3 and Section 2.2.4.

#### 2.2.1. Data Sources and Preprocessing

The primary data used in this study included the NDVI, total precipitation (PRE), mean temperature (TEM), minimum temperature (TMN), maximum temperature (TMX), total solar radiation (SR), potential evapotranspiration (PET), actual evapotranspiration (AET), vapor pressure deficit (VPD), soil moisture (SM), annual aridity index (AI), relative humidity (RHU), grazing intensity (GI), and artificial nighttime light (NTL). NDVI was derived from the PKU GIMMS NDVI product from Peking University (https://zenodo.org/records/8253971, accessed on 15 January 2025) [15]. This dataset provides a temporally consistent multi-decadal record and has been harmonized to reduce inter-sensor inconsistencies, which is essential for assessing long-term changes [15]. Although higher-resolution products (e.g., MODIS, Landsat, Sentinel) are available, their temporal coverage does not fully match the study period or requires extensive cross-sensor harmonization.

The five ecosystem types (cropland, forest, shrubland, grassland, and wetland) in this study were defined directly using the original classes of the China Land Cover Dataset (CLCD) developed by Yang and Huang (https://zenodo.org/records/12779975, accessed on 15 January 2025) [16]. The distribution ranges of these five ecosystems changed between 1985 and 2020. To ensure that ecosystem-wise comparisons were not confounded by land-cover conversions, we conducted ecosystem-specific analyses only within “stable pixels”, i.e., pixels whose CLCD ecosystem class remained unchanged throughout the study period. These stable pixels were used as masks for cropland, forest, shrubland, grassland, and wetland in all ecosystem-stratified statistics.

The TEM, TMN, TMX and PRE data were from the National Earth System Science Data Center (https://www.geodata.cn/main/face_science_detail?typeName=face_science&guid=164304785536614, accessed on 15 January 2025) [17]. The SR data were derived from the Geographic Remote Sensing Ecological Network Platform (https://www.gisrs.cn/?da-ta_89/2eed451f-e4c1-415f-a32c-20bfb16644cf.html=, accessed on 15 July 2025) [18]. The PET, AET, SM and VPD data were obtained from the Climatology Lab NSF National Center for Atmospheric Research (https://www.climatologylab.org/terraclimate.html, accessed on 15 July 2025) [18]. The RHU data were from the National Cryosphere Desert Data Center (https://www.ncdc.ac.cn/portal/metadata/21691d03-bef2-4800-924e-5614e7268b87, accessed on 15 July 2025) [19,20,21]. The AI data were from the National Earth System Science Data Center (https://www.geodata.cn/main/face_science_detail?id=62494&guid=188606016270010, accessed on 15 July 2025) [17,22,23,24]. The GI data were from the National Ecosystem Science Data Center (https://www.nesdc.org.cn/sdo/detail?id=672aea607e28174998e63234, accessed on 15 July 2025) [25]. The NTL data were obtained from the National Tibetan Plateau Data Center (https://data.tpdc.ac.cn/zh-hans/data/e755f1ba-9cd1-4e43-98ca-cd081b5a0b3e, accessed on 15 July 2025) [26,27]. Detailed information is displayed in Table 1.

To minimize abnormal fluctuations caused by cloud cover or other weather-related factors, the maximum value composite method was used to aggregate biweekly NDVI data to a monthly scale. Annual data required for subsequent analyses were derived from monthly data when the original temporal resolution was monthly. To ensure consistent spatial resolution across all of the datasets, the nearest neighbor interpolation method was used to resample the data to 1 km × 1 km. Although all datasets were resampled to 1 km for spatial alignment, the effective spatial detail of the NDVI-based vulnerability index is constrained by the original 8 km NDVI resolution. Therefore, spatial interpretation is most appropriate at regional scales rather than at fine local micro-topographic scales. The World_Geodetic_System_1984 coordinate system was uniformly adopted for all of the spatial data.

#### 2.2.2. Vulnerability Assessment Model

We used a sliding-window approach to analyze temporal trends in vulnerability while reducing model uncertainty from extreme climatic or other sudden events. This method divides the entire time series into contiguous, fixed-length segments and advances the window stepwise across the record. The approach is widely used in long-term studies of vegetation dynamics [28,29,30]. Here, we applied a five-year sliding window with a one-year step to monthly NDVI, temperature, and precipitation data from 1983 to 2022. Starting in 1983 and advancing annually to 2022, this produced 36 overlapping five-year periods (1983–1987, 1984–1989, …, 2018–2022), each comprising 60 months.

We assessed ecological vulnerability using the widely adopted exposure–sensitivity–resilience framework [31,32]. Exposure denotes the degree of climatic disturbance that terrestrial ecosystems may undergo [33]. Sensitivity indicates how strongly an ecosystem responds to a given disturbance [34,35]. Resilience denotes an ecosystem’s capacity to recover to its original state after a disturbance [36]. Following Li et al. [37], we combined these three components into a composite ecological vulnerability index (VI). A detailed description of the VI assessment is provided in Jiao et al. [14] and in the Supplementary Materials. The calculation is detailed in Equations (1)–(5):

(1) $${NDVI}_{t}=\alpha \times {Temp}_{t}+\beta \times {Pre}_{t}+\gamma \times {NDVI}_{t-1}+\varepsilon_{t}$$

(2) EI=α+β

(3) $$SI=\alpha \times T_{-norm}+\beta \times P_{-norm}$$

(4) RI=1−γ

(5) $$VI=\sqrt{\frac{EI\times SI}{1+RI}}$$

In Equations (1)–(5), ${NDVI}_{t}$ and ${NDVI}_{t-1}$ are the monthly standardized NDVI anomalies at time t and t−1. ${Temp}_{t}$ and ${Pre}_{t}$ are the monthly standardized temperature and precipitation anomalies at time t. α, β, and γ are the fitting coefficients, and $\varepsilon_{t}$ is the residual term. Here, monthly NDVI, temperature, and precipitation data within each five-year moving window were standardized using z-scores in order to ensure comparability between model coefficients. The coefficients α, β, and γ were normalized to a 0–1 range using min-max scaling. This would ensure scale-invariant indices, free from bias due to variable magnitudes. $T_{-norm}$ and $P_{-norm}$ represent the mean normalized temperature and precipitation within each five-year window, respectively. VI is the Ecological Vulnerability Index; EI is the Exposure Index; SI represents the Sensitivity Index; and RI represents the Resilience Index.

#### 2.2.3. Analysis of Interannual Variation in Vulnerability

We first computed the coefficient of variation (CV) for VI at the pixel scale for 1985–2020. Then, we characterized the interannual VI dynamics using three methods: simple linear regression, piecewise linear regression, and the Pettitt test. We screened the results across methods using t-tests, *p*-values, and the Akaike Information Criterion (AIC). AIC is a statistical method used for model selection. A lower AIC value indicates a better model as it balances goodness of fit (measured by the likelihood) with the model’s complexity (the number of parameters). This helps prevent overfitting. If all of the methods yielded non-significant *p*-values (*p* ≥ 0.05), the pixel was classified as having no significant change trend in VI. If only one method produced *p* < 0.05, we adopted that method’s change pattern. If multiple methods produced *p* < 0.05, we selected the model with the lowest AIC value as the best representation. If the models yielded equal AIC values, we prioritized the simpler model (e.g., a linear model over a piecewise linear model).

To ensure the robustness of the results, we re-evaluated the trend for pixels where the VI trend turning point identified by piecewise linear regression or the Pettitt test fell within five years of either the start year (1985) or the end year (2020). For each such pixel, we fitted a simple linear regression. If the *p*-value was significant, we classified the pixel as exhibiting a linear trend; otherwise, we classified it as showing no significant trend. We then summarized the change patterns in VI across the TENS into three classes: no significant trend, a linear trend, and an abrupt trend with a turning point. Linear trends were further classified into two types: significant increases and significant decreases. Abrupt trends were categorized as a significant increase followed by a significant decrease, or a significant decrease followed by a significant increase.

#### 2.2.4. Analysis of Factors Influencing Interannual Variability in Vulnerability

This study used the eXtreme Gradient Boosting (XGBoost) model, together with Shapley Additive exPlanations (SHAP) and Partial Dependence Plots (PDP), to systematically evaluate how climatic factors and human activities drive interannual variability in ecosystem vulnerability. XGBoost is an ensemble algorithm derived from Gradient Boosting Decision Trees (GBDT). The model introduces regularization terms and leverages second-order derivative information from GBDT, thereby improving predictive accuracy and computational efficiency. For these reasons, XGBoost has been widely adopted in data-intensive applications [38,39].

In this study, we preselected 13 candidate influencing factors: PRE, PET, AET, AI, VPD, RHU, SM, TEM, TMX, TMN, SR, GI, and NTL. To minimize the effects of multicollinearity on model stability, we first computed variance inflation factors (VIFs) for each variable and excluded those with a VIF ≥ 10. The remaining variables were used as input features for the XGBoost model. During model development, 80% of the samples were allocated to the training set and 20% to the test set. We optimized model hyperparameters using a grid search to identify the best parameter set. Model performance was assessed using the coefficient of determination (R^2^), the mean squared error (MSE), the root mean squared error (RMSE), and the mean absolute error (MAE).

To improve the interpretability of the XGBoost model, we applied the SHAP framework proposed by Lundberg et al. to quantify each feature’s contribution to model outputs [40]. SHAP is grounded in cooperative game theory and computes the marginal contribution of every feature subset to a prediction. This yields a consistent, additive attribution of the model’s decision process. To illustrate how individual factors influence interannual changes in ecosystem vulnerability, we also produced partial dependence plots (PDPs) for the four variables with the highest importance scores. PDPs demonstrate the average effect of varying the target variable on predicted VI while holding other variables constant at their mean values, thereby revealing potential nonlinear responses and threshold behavior. In this study, all of the raster processing, statistical analyses, and graphics were performed in R 4.4.1 [41], and spatial pattern maps were created in ArcGIS 10.8 [42].

## 3. Results

### 3.1. Interannual Variation in Vulnerability

**Figure 3:**
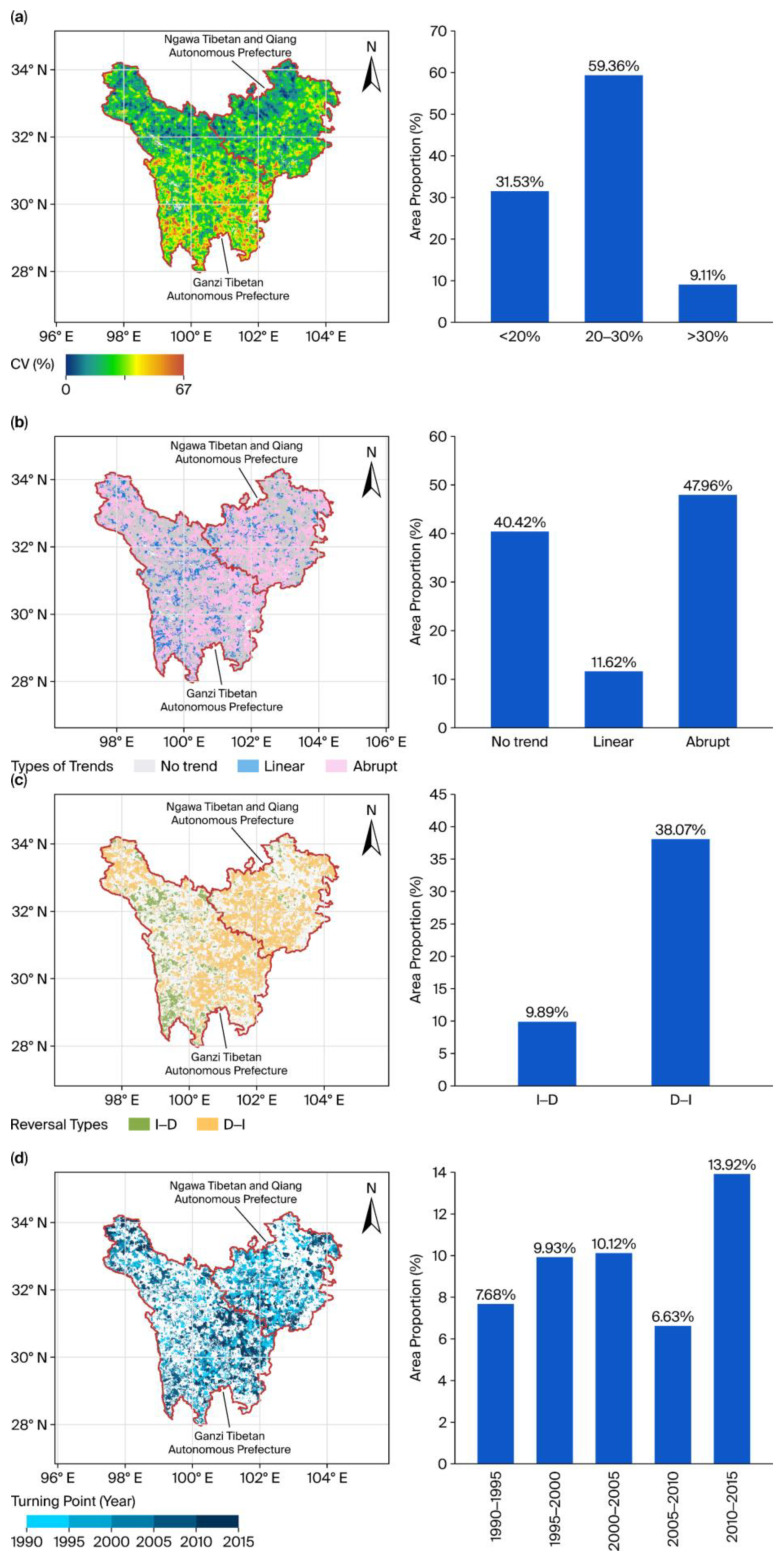
Spatial patterns of the change trend for VI. (**a**) Spatial pattern of the coefficient of variation (CV) for VI; (**b**) Spatial patterns of different types of VI change trends; (**c**) Spatial patterns of different turning points in VI change trends; (**d**) The spatial pattern of the years when VI trends shifted (interval from 1985 to 2020). No trend, Linear, and Abrupt denote that VI shows no significant trend, exhibits a linear trend, and presents a significant turning point from 1985 to 2020, respectively. I–D indicates a shift from a significant increase to a significant decrease, and D–I indicates a shift from a significant decrease to a significant increase.

The spatiotemporal dynamics of VI in TENS from 1985 to 2020 exhibited marked spatial heterogeneity (Figure 3 and Figure S1). The CV for VI displayed a latitudinal gradient, increasing progressively from north to south (Figure 3a). Area statistics showed that regions with moderate variation (CV between 20% and 30%) accounted for the majority of TENS, comprising 59.36% of the total area. Regions with low variation (CV < 20%) were primarily located in the north and accounted for 31.53% of the area. Regions with high variation (CV > 30%) were concentrated along the southern margin and accounted for 9.11% of the total (Table S1).

The temporal dynamics of VI across space were categorized into three types: no significant trend, a linear trend, and an abrupt trend (Figure 3b). Across TENS, 47.96% of the area exhibited an abrupt change trend, indicating a pronounced nonlinear shift in ecosystem vulnerability in nearly half of the region during the study period. By contrast, 40.42% of the area showed no significant trend. Areas with a single significant linear trend comprised 11.62% of TENS. Of these, regions with a linear increase in VI—reflecting increasing vulnerability—covered 6.96% of TENS, slightly more than the 4.66% that showed a linear decrease in VI (Table S1).

For regions where the VI trend showed a turning point, we further examined the spatial distribution of turning modes and their timing of occurrence (Figure 3c,d). The transition from a significant decreasing trend to a significant increasing trend (type D–I) was predominant (Figure 3c), with 37.89% of the total TENS, representing roughly 80% of the area showing any reversal in trend. In contrast, the reverse transition—from a significantly increasing trend to a significantly decreasing trend (type I–D)—covered only 9.90% (Table S1). Regarding the timing of turning points (Figure 3d), these occurred between 1990 and 2015, with a peak frequency between 2010 and 2015. During that interval, areas with turning points accounted for 13.92% of TENS, a proportion notably larger than earlier intervals such as 2000–2005 (9.81%) and 1995–2000 (9.74%) (Table S1).

**Figure 4:**
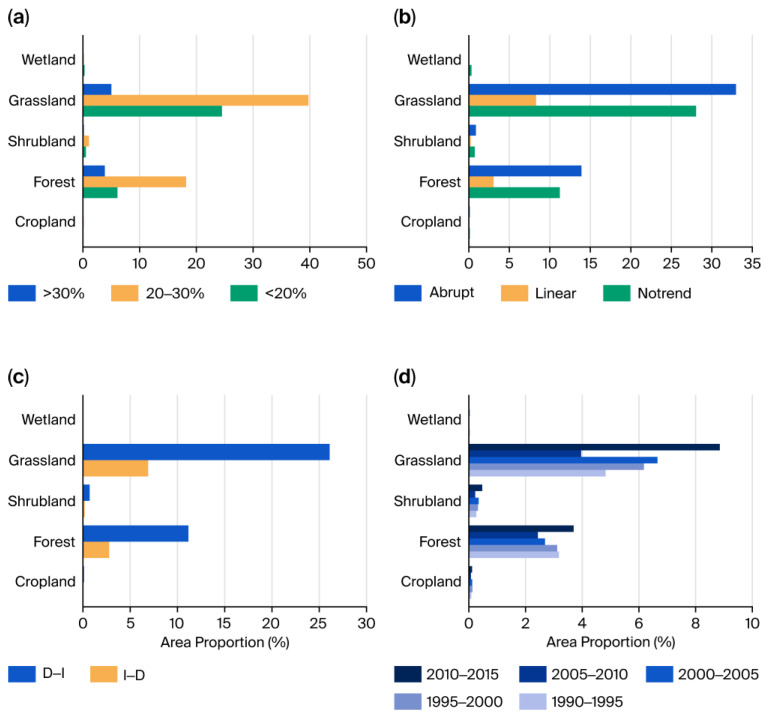
Interannual variation in VI across various ecosystems. (**a**) Coefficient of variation for VI; (**b**) Different types of VI change trends; (**c**) Different turning patterns of VI change trends; (**d**) The year when the VI change trends reversed. CV indicates the coefficient of variation for VI during the period from 1985 to 2020. No trend, Linear, and Abrupt denote that VI shows no significant trend, exhibits a linear trend, and presents a significant turning point from 1985 to 2020, respectively. I–D indicates a shift from a significant increase to a significant decrease, and D–I indicates a shift from a significant decrease to a significant increase.

The temporal dynamics of VI across different ecosystems exhibited significant variation (Figure 4). The overall change pattern in TENS was dominated by the widely distributed grassland and forest ecosystems. In terms of CV (Figure 4a), grassland and forest ecosystems showed similar moderate fluctuations. The CVs of most areas in these two ecosystems ranged from 20% to 30%, accounting for 39.77% and 18.21% of the total TENS area, respectively (Table S1). The next most common category was low variability (CV < 20%). Areas with high variability (CV > 20%) had relatively limited proportions. Due to their smaller areas, the CV distributions of shrubland, cropland, and wetland contributed relatively little to the overall regional characteristics.

In terms of change-trend types (Figure 4b), the various ecosystems exhibited distinct characteristics. Natural or semi-natural ecosystems such as grassland, forest, and shrubland showed higher dynamic instability. These ecosystems were internally dominated by “abrupt change trends”. Areas experiencing abrupt changes accounted for 32.98%, 13.91%, and 0.87% of the total TENS area, respectively (Figure 4b, Table S1). Cropland ecosystems under strong human intervention and wetland ecosystems were relatively stable and were characterized as “no significant change trend”.

Further analysis of the shift patterns in abrupt change areas revealed the following (Figure 4c). In all ecosystems dominated by “abrupt change trends”, the proportion of the D–I type was significantly higher than that of the I–D type. Areas undergoing D–I type transitions in grassland and forest accounted for 26.08% and 11.15% of the total TENS area, respectively. In contrast, the I–D type accounted for only 6.89% and 2.75% (Figure 4c, Table S1).

Regarding the timing of shift occurrence, different ecosystems showed high synchrony (Figure 4d). Overall, although the shift years across all of the ecosystem types were widely distributed between 1990 and 2015, the period 2010–2015 was the most intensive for ecosystem shifts (Table S2). The areas undergoing shifts in grasslands and forests during this period were 9.60% and 4.02%, respectively, significantly higher than in other periods. The shifts in the proportions of shrubland, cropland, and wetland during 2010–2015 were also slightly higher than in earlier periods. In contrast, the shift areas during 1990–1995 and 2005–2010 were relatively small. The periods of 1995–2000 and 2000–2005 showed moderate levels.

### 3.2. Analysis of Factors Influencing the Interannual Variation in Vulnerability in the TENS

Among the 13 initially selected potential influencing factors, we first eliminated those with high multicollinearity through VIF tests. We ultimately identified eight factors as input variables for the XGBoost model; these were PRE, AET, VPD, SM, TMX, TMN, SR, and GI. The model showed high fitting accuracy, with relatively low RMSE and MAE values (Table S2). This indicated that the constructed XGBoost model could reliably characterize the factors influencing the temporal dynamics of VI.

**Figure 5:**
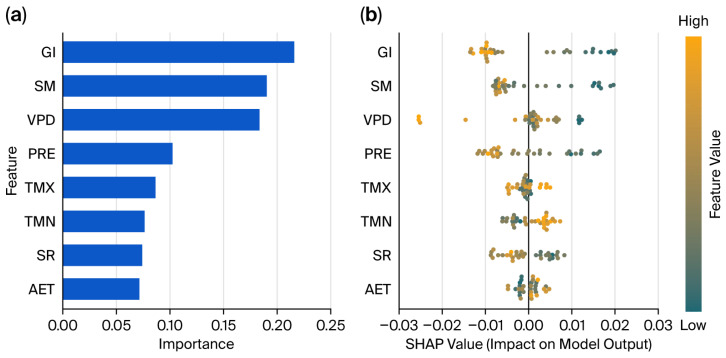
Ranking of the importance of factors influencing interannual variation in VI in TENS (**a**) and SHAP summary plots (**b**).

The feature importance ranking of the XGBoost model showed that GI, SM, and VPD were the three most important factors affecting interannual variation in VI (Figure 5a). Their relative importance values were 21.58%, 19.03%, and 18.35%, respectively. The cumulative contribution of these three factors approached 60% (Table S3). Although PRE is the primary source of water for ecosystems, its contribution rate was only 10.24%. This was significantly lower than those of SM and VPD (Table S3). Heat and energy factors such as TMX, TMN, and SR, as well as AET, showed relatively low independent contribution rates. These factors functioned more as background environmental variables, contributing to the variation in VI by synergistically regulating ecosystem processes.

We used SHAP summary plots to link sample values (color) with SHAP effects (position) in order to assess the direction and magnitude of each driving factor on the variation in VI (Figure 5b). High GI samples (orange) clustered in the negative SHAP region, while low GI samples (blue) appeared in the positive region (Figure 5b). This pattern indicates that, across the study area’s environmental gradient, higher grazing intensity exerted a strong negative effect on VI, thereby reducing ecosystem vulnerability. At low grazing intensity, the dispersed blue points imply that GI alone does not dominate the variation in VI, while background environmental conditions and interactions with other factors shape VI. SM and PRE showed analogous patterns (Figure 5b). Low-value samples (blue) were concentrated in the positive SHAP region, indicating that water deficit is a primary risk factor increasing ecosystem vulnerability, while high-value samples (orange) fell in the negative region, confirming that improved moisture conditions mitigate vulnerability.

The distribution of VPD and its SHAP values shows a clear “baseline–threshold” pattern (Figure 5b). Most sample points cluster near the baseline, where SHAP values equal zero. This pattern indicates that, in most cases, atmospheric water demand in this region remains within a range suitable for vegetation. The natural variability in VPD does not substantially disturb ecological vulnerability. However, the distribution’s long tail shows different behavior. Once VPD exceeds a specific threshold, SHAP values rapidly deviate from zero. VPD then becomes a decisive driver of vulnerability. This result indicates that VPD has a triggering effect on ecosystem VI, primarily under extreme conditions. VPD does not act as a continuous pressure factor, like GI and SM do, and instead functions as a potential risk factor. Sample points for TMX, TMN, SR, and AET were tightly concentrated in low SHAP value ranges (Figure 5b). This pattern further supports their roles as auxiliary regulating factors.

**Figure 6:**
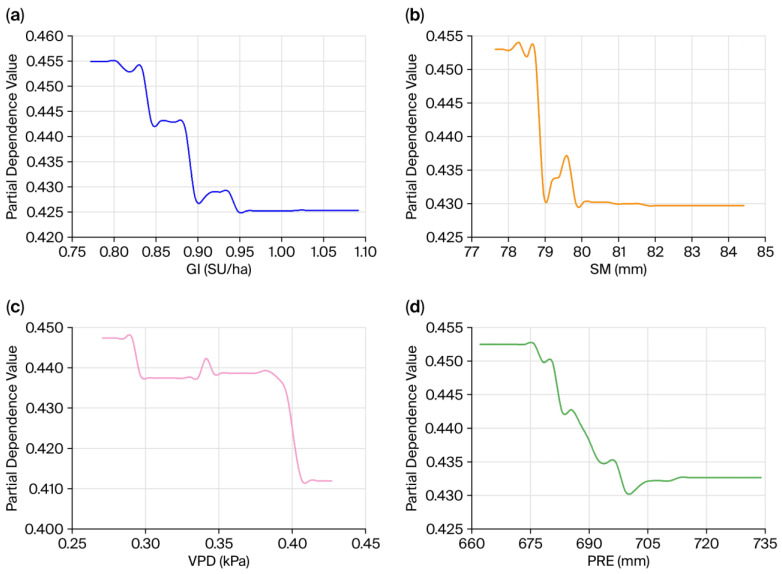
Partial dependence plots of the top four variables influencing interannual variation in VI in TENS. (**a**) GI; (**b**) SM; (**c**) VPD; (**d**) PRE.

The PDPs of the four most important influencing factors showed that all of the factors exerted significant nonmonotonic or stage-dependent effects on interannual variation in VI (Figure 6). The response of VI to GI shows a nonlinear pattern with a clear threshold (Figure 6a). When GI is below 0.90 SU/ha, VI declines in a stepwise manner with increasing grazing intensity. When GI exceeds the critical threshold of 0.90 SU/ha, the VI trend reverses. VI shows a slight increase, and then stabilizes at 0.95 SU/ha. The response of VI to SM is similar to that for GI and shows a strong threshold effect (Figure 6b). When SM is below 79 mm, VI remains high. This pattern indicates that water deficit is the primary constraint on the ecosystem in this region. As SM increases and exceeds 79 mm, VI decreases sharply. This result suggests a strong marginal effect of water supply in alleviating ecosystem vulnerability. When SM exceeds 80 mm, the VI curve rapidly enters a low value plateau.

Unlike common perceptions for arid regions, VPD and VI in the study area showed a distinct stepwise negative relationship (Figure 6c). At low VPD levels between 0.27 and 0.29 kPa, VI remains high. This pattern may be related to the cold, humid alpine environment, where very low atmospheric water demand is often associated with low temperatures or insufficient radiation. These conditions suppress the physiological activity of the vegetation. As VPD increases, VI shows two abrupt stepwise declines. When VPD exceeds 0.29 kPa, VI rapidly decreases to about 0.44; above 0.39 kPa, VI further declines to a minimum of about 0.41 and then remains stable.

The effect of precipitation on VI (Figure 6d) is highly consistent with that of SM. This result further confirms the dominant role of the water supply. When precipitation is less than 705 mm, increases in precipitation are significantly negatively correlated with VI. This relationship indicates that water input effectively reduces ecosystem stress. When precipitation exceeds 705 mm, the slope of the curve approaches zero.

### 3.3. Comparative Analysis of Factors Influencing Interannual Variability in Vulnerability Across Ecosystem Types

**Figure 7:**
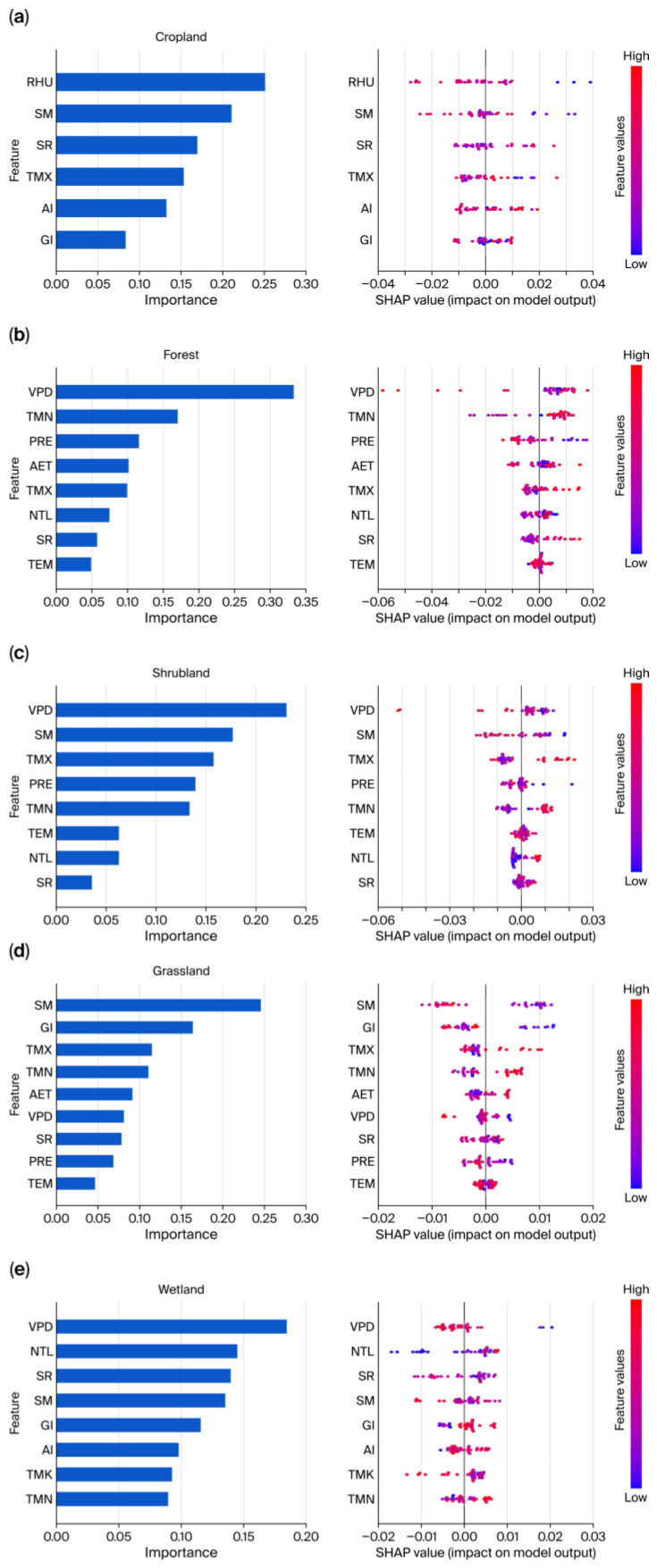
Importance ranking and SHAP summary plots of factors influencing interannual variation in VI across different ecosystems. (**a**) Cropland ecosystems; (**b**) forest ecosystems; (**c**) shrubland ecosystems; (**d**) grassland ecosystems; and (**e**) wetland ecosystems.

**Figure 8:**
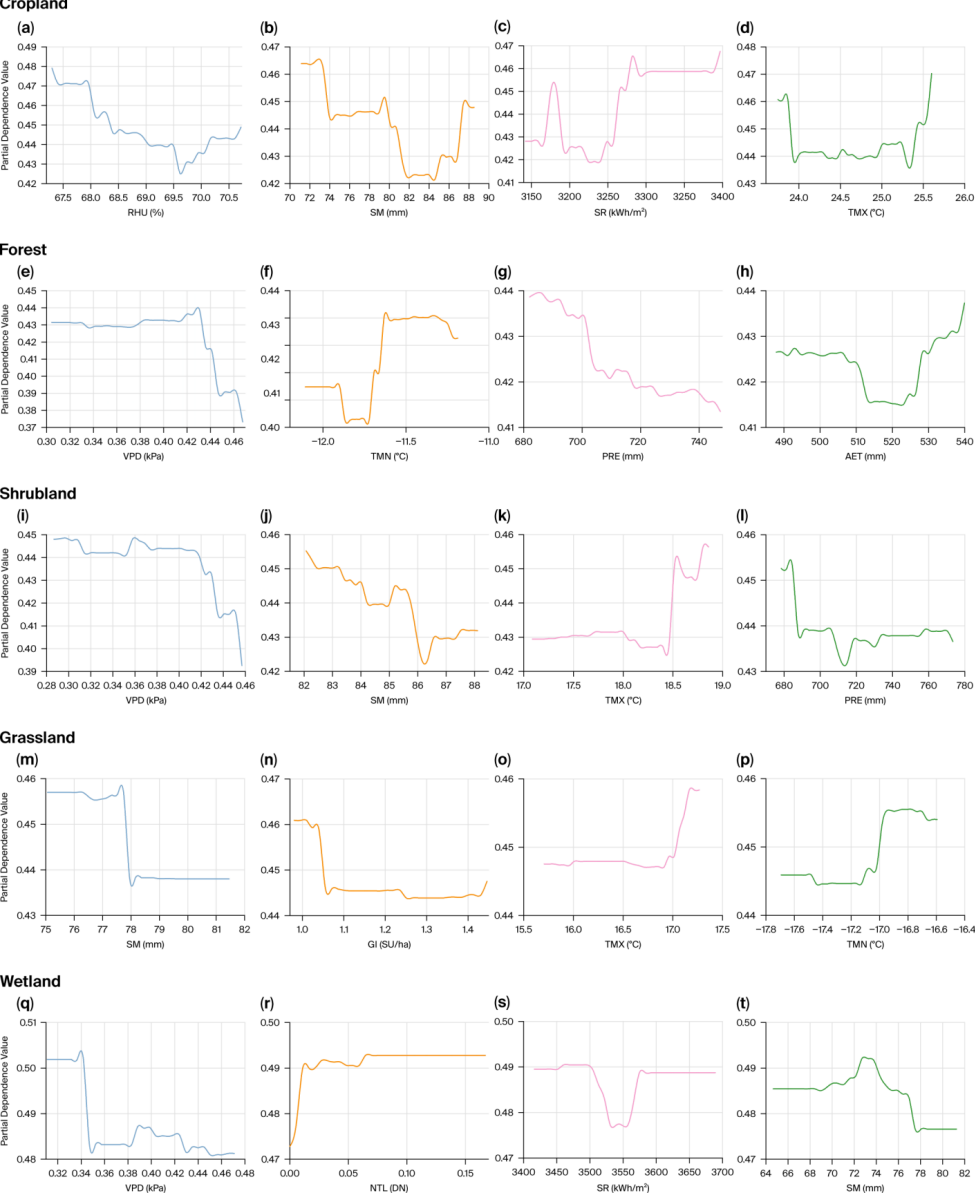
PDPs of the four primary factors influencing interannual variation in VI across different ecosystems. (**a**–**d**) Cropland ecosystems; (**e**–**h**) Forest ecosystems; (**i**–**l**) Shrubland ecosystems; (**m**–**p**) Grassland ecosystems; (**q**–**t**) Wetland ecosystems.

To minimize multicollinearity effects on model performance, we first conducted VIF tests on 13 preselected candidate predictors for each ecosystem type. The variables that passed screening served as inputs to the XGBoost model and were used to analyze drivers of interannual VI variability (Figure 7, Table S3). Model fit was strong across all ecosystem types (Table S2). R^2^ values ranged from 0.60 to 0.99, while RMSE and MAE remained low. The results indicate that VI in cropland ecosystems is primarily controlled by the water supply and energy conditions (Figure 7a). RHU and SM were the dominant drivers, contributing 25.11% and 21.05%, respectively. SR and TMX followed, with contribution rates of 16.95% and 15.31%, respectively (Table S3). The SHAP summary plot shows that samples with high RHU and SM values clustered in the negative SHAP region, indicating that sufficient water supply helps reduce cropland vulnerability. PDP analyses further revealed threshold ranges for optimal vegetation growth (Figure 8a–d). VI responses to RHU and SM followed a V-shaped, nonlinear pattern, revealing clear water thresholds (RHU at about 69.5% and SM at about 71–82 mm). Values below these thresholds produced sharp increases in vulnerability due to water deficit, while excess water can cause waterlogging or other adverse effects that increase VI (Figure 8a,b). The U-shaped responses of VI to SR and TMX indicate that both excessively high and low radiation and temperature deviate from the ecological optima for crop growth. These deviations, in turn, increase ecosystem vulnerability (Figure 8c,d).

The VI of forests is primarily influenced by atmospheric water demand and temperature (Figure 7b). VPD contributes 33.28%, making it the principal determinant of forest vulnerability (Table S3). The substantial heterogeneity in the SHAP value distributions and the PDP curves indicates that VPD has an overall negative association with VI, becoming particularly pronounced when VPD exceeds 0.42 kPa, leading to a sharp decline in VI (Figure 8e). TMN was the second most important factor, contributing 17.02%. High TMN values are generally found within the positive SHAP region, a pattern confirmed by the PDP curves (Figure 8f). Extremely low TMN values below −11.9 °C markedly increase forest vulnerability, and extremely high TMN values above −11.5 °C produce a similar effect. In addition, the negative influence of PRE (Figure 8g) and the U−shaped response to AET (Figure 8h) further underscore these dynamics. Together, these results highlight the central role of water and heat balance in shaping the vulnerability of forest ecosystems.

Shrub ecosystems are situated in the transition zone between forests and grasslands. Their VI is primarily controlled by atmospheric and soil moisture (Figure 7c). The contribution rates of VPD and SM were 23.05% and 17.70%, respectively (Table S3). The influence of VPD on shrubs is similar to that observed in forests (Figure 8i), reflecting comparable physiological strategies among woody plants. Shrubs also exhibit a pronounced threshold response to SM, with a turning point near 86 mm (Figure 8j). When SM falls below this threshold, the VI decreases significantly with increasing SM. When SM exceeds this threshold, VI shows an upward trend, suggesting that excessively wet conditions may suppress root respiration and induce physiological stress in shrubs. Shrub VI is likewise highly sensitive to TMX (Figure 8k), remaining low and variable when TMX is below 18.4 °C, while rising sharply above this value. Increasing PRE can partially mitigate shrub vulnerability (Figure 8l). The VI decreases significantly as PRE increases when PRE is less than approximately 715 mm. This alleviation effect tends to saturate when PRE exceeds this value.

When comparing cropland, forest, and shrub ecosystems, the variation in grassland VI was driven primarily by the combined effects of SM and GI (Figure 7d), with contribution rates of 24.54% and 16.38%, respectively (Table S3). PDPs revealed an apparent threshold effect of SM on grassland VI (Figure 8m). When SM was below 78 mm, water limitation allowed VI to remain high. Once SM exceeded 78 mm, VI dropped sharply and then stabilized. Regarding human activities, GI exhibited a nonlinear negative relationship with VI (Figure 8n). The VI decreased significantly with increasing GI, provided that GI was controlled at 1.21 SU/ha. The VI fluctuated at a lower level when GI exceeded this value. TMX and TMN were the third- and fourth-most important factors, contributing 11.47% and 11.05%, respectively (Table S3). Both variables markedly increased vulnerability once they exceeded specific thresholds, namely, −18.4 °C for TMX and −17 °C for TMN (Figure 8o,p).

Wetland ecosystems exhibited more complex responses to environmental factors. The variation in VI was jointly influenced by VPD, NTL, SR, and SM (Figure 7e). These four factors showed comparable relative importance, with contribution rates of 18.45%, 14.49%, 13.96%, and 13.53%, respectively (Table S3). NTL serves as an indicator of the intensity of human activity. Its PDP curve shows that even weak anthropogenic disturbance can cause a rapid increase in wetland VI, which then remains elevated (Figure 8r). This indicates that wetland ecosystems are susceptible to human activities.

Regarding natural factors, VI showed a complex nonlinear response to VPD (Figure 8q). VI is maintained at a high level at low VPD. As VPD increases to approximately 0.35 kPa, VI drops sharply. Subsequently, VI shows an increasing trend as VPD continues to rise. However, after VPD exceeds 0.39 kPa, VI gradually declines with increasing VPD. VI exhibits a V-shaped response to SR (Figure 8s). The optimal stability point occurs at approximately 3550 kWh/m^2^, where VI reaches its minimum value. The influence of SM on VI displays a unique unimodal pattern (Figure 8t). VI reaches its peak at SM ≈ 73 mm. Beyond 73 mm, VI declines sharply.

## 4. Discussion

### 4.1. Non-Linear Evolution of Vulnerability

Our analysis indicates that the temporal trends in vulnerability across TENS are spatially heterogeneous rather than uniform, static, or linear, and instead demonstrating significant stage-specific characteristics. Unlike previous studies that primarily focused on static vulnerability patterns and linear trends [3,8,43,44,45], our results reveal that nearly half (47.96%) of the areas within TENS experienced significant turning points in vulnerability. These turning points largely occurred between 2010 and 2015. It is noteworthy that 37.89% of the regions in TENS showed a reversal from “significant decrease” to “significant increase” in vulnerability (D–I). These areas are primarily distributed across the eastern regions of Ngawa and Ganzi Prefectures. This suggests that the sustainability of ecological restoration in these regions faces considerable challenges.

The shift from ecological improvement to degradation in some regions of TENS could be attributed to the combined effects of “ecological engineering saturation” and “climate fluctuations”. In the early part of the study period, the reduction in vulnerability primarily benefited from government-implemented ecological restoration projects, such as “Grain for Green” [46,47] and “Desertification Control” [48]. This is consistent with the ecological improvement trend found by Zhang et al. in the Yellow River Basin [9]. However, the reversal after 2010 warns us that relying solely on vegetation quantity restoration (such as afforestation and grass planting) to assess vulnerability may be misleading as this factor may have approached the ecosystem’s carrying capacity [49]. According to the “ecosystem stability and regime shift” theories [50], increased evapotranspiration demand induced by climate change, combined with local overgrazing, may offset the positive effects of early ecological engineering, thereby decreasing ecosystem resilience [51,52]. In addition, extreme climate events (such as seasonal droughts) occurring in the southeastern margin of the Tibetan Plateau during 2010–2015 may have acted as triggering factors [53], inducing this shift. This indicates that the current ecological management strategy of TENS urgently needs to transform from “quantity expansion” to “quality improvement” and “risk management”.

### 4.2. Coupling Driving Mechanisms and Threshold Effects of “Human Disturbance–Soil Moisture–Atmospheric Water Demand”

This study employed an XGBoost–SHAP model to quantify the nonlinear effects and threshold responses of driving factors on TENS vulnerability. This approach addressed the limitations of traditional linear regression methods. The results showed that GI, SM, and VPD were the three core factors driving interannual variation in vulnerability. Their cumulative contribution rate approached 60%. This indicated that the coupling process of “human disturbance–soil moisture–atmospheric water demand” played a dominant role in the dynamics of ecological vulnerability in this region.

The impact of grazing showed that TENS vulnerability declines as grazing intensity (GI) increases when GI is less than 0.90 SU/ha. This may be attributed to two principal aspects. One is that grazing areas are often located in high-quality grasslands with high baseline productivity and strong resilience, factors that confer a “baseline advantage” [54]. Another is that moderate grazing and trampling remove litter, promote seed germination, and enhance nutrient cycling, thereby stimulating compensatory vegetation growth [55]. However, once grazing intensity exceeds the threshold of 0.90 SU/ha, these positive effects disappear, and vulnerability either stabilizes or recovers. This phenomenon aligns with the characteristics of the “intermediate disturbance hypothesis” [56]. The hypothesis suggests that moderate grazing intensity can increase grassland biodiversity and reduce ecosystem vulnerability [57]. However, excessive grazing pressure offsets these positive effects and produces diminishing marginal returns, increasing vulnerability when the ecological carrying capacity is exceeded. This finding alters the traditional one-sided perception that “grazing equals disturbance”. The finding underscores the critical role of determining region-specific stocking rate thresholds to maintain ecosystem health [58].

The influence of water conditions is manifested by the dominance of SM over PRE. This indicates that directly available “effective water” is more important for vegetation growth than total atmospheric precipitation [59]. Regarding the impact on TENS vulnerability, SM exhibits a clear “threshold point” (approximately 79–80 mm). Below this range, water deficit act as a bottleneck limiting ecosystem development. Above this range, water is no longer a limiting factor [60]. This phenomenon also explains why ecosystems remain highly vulnerable in some years with abundant precipitation. This is likely due to differences in soil water retention capacity, which cause a decoupling of water availability [61].

Regarding the influence of atmospheric water demand, this study shows a unique stepwise negative correlation between VPD and vulnerability when VPD is below 0.39 kPa. This is markedly different from the common understanding that “high VPD triggers physiological drought” in arid and semi-arid regions [62,63,64]. In the alpine humid/semi-humid region of northwestern Sichuan, a lower VPD is usually accompanied by low temperatures, high cloud cover, and low radiation levels. Conversely, a higher VPD often corresponds to sufficient solar radiation and suitable temperatures. This helps remove energy limitations on alpine vegetation and enhances photosynthetic efficiency [65]. Therefore, within the environmental gradient of this study area, VPD acts more as an indicator of “hydrothermal synergy” than as a simple water-stress factor.

### 4.3. Differential Response Mechanisms Across Different Ecosystem Types

Differences in the structure and function among ecosystem types determine their response strategies to environmental driving factors, and these are highly heterogeneous. Grassland ecosystems are most vulnerable to SM and GI. This is because herbaceous plants, unlike woody plants, have shallow root systems and cannot access deep groundwater, thus showing extremely high dependence on the fluctuations in soil moisture [66]. In contrast, grasslands serve as the primary resource for pastoral development and are directly under pressure from human activities [67]. This explains to some extent why grasslands are the main areas where D–I type trend reversals (vulnerability rebounds) occur.

VPD and TMN are the most important drivers of forest ecosystems. This is primarily because the well-developed root systems of woody plants provide a specific buffering capacity against short-term soil drought [68]. However, the large exchange of water vapor between tree canopies and the atmosphere makes them highly sensitive to VPD [64]. The PDP analysis showed that excessively low TMN (below—11.9 °C) significantly exacerbated the vulnerability of forest ecosystems. This indicates that low-temperature frost damage or shortened growing seasons are important factors limiting the growth of high-altitude forest ecosystems. Forests can benefit from moderate increases in temperature, as they extend growing seasons [69]. Excessively high TMN (above—11.5 °C) sharply increases ecosystem vulnerability. This suggests that extremely high temperatures pose new physiological stressors to forest ecosystems [70]. Shrubland ecosystems are primarily affected by VPD and SM, reflecting the combined physiological responses of woody and herbaceous plants [71].

Cropland ecosystems are regulated by moisture conditions, with RHU and SM being the dominant factors. The PDP analysis showed that VI exhibited a “V–shaped” response to both RHU and SM. This indicates that both drought and excessive wetness can intensify the vulnerability of cropland ecosystems. This finding aligns with the delicate dependence of cropland ecosystems on moisture, where both excess and deficiency are detrimental [72,73].

Wetland ecosystems reflect a strong imprint of human activities. The high contribution rate (NTL: 14.49%) directly reflects disturbances to wetland peripheries caused by human activities such as urban expansion and tourism development. The PDP analysis shows that even very low levels of human activity can lead to a sharp increase in wetland vulnerability. This highlights the extreme fragility of wetlands as “ecologically sensitive areas” [74]. It also suggests the need to establish strict wetland protection systems [75].

### 4.4. Research Limitations and Prospects

Although this study used an XGBoost–SHAP framework to reveal nonlinear responses of interannual TENS vulnerability to climate and human activities, the approach has several limitations.

(1) Vegetation responses to climate variability may involve time-lag and cumulative effects. However, lagged responses were not explicitly incorporated in the present framework, which may lead to under- or over-estimation of vulnerability in some areas, especially in ecosystems with delayed recovery.
(2) The VI relies primarily on NDVI as the core spatial input. Although all variables were resampled to a common 1 km grid for spatial alignment, the effective spatial detail of the NDVI-based VI remains constrained by the original 8 km NDVI resolution. This scale mismatch may introduce mixed-pixel effects and smooth fine-scale ecological heterogeneity, which is particularly relevant in complex mountainous terrain and mosaic landscapes. Therefore, the spatial interpretation is most robust at regional rather than micro-topographic scales.
(3) Ecosystem-wise analyses were performed for the five ecosystem types as defined by the original CLCD classes, and only within pixels whose ecosystem class remained unchanged during 1985–2020. This strategy reduces confounding from land–cover conversions, but it also means that our ecosystem-specific results primarily represent vulnerability dynamics within persistent ecosystem backgrounds. Areas experiencing ecosystem transitions were excluded, and the coupled effects of land–cover change and vulnerability trajectories were not quantified.
(4) The study is largely based on remote sensing and gridded datasets, and independent ground-based validation was not available. Exclusive reliance on remote observations can introduce interpretation errors when remotely sensed signals do not fully reflect on-the-ground ecosystem conditions. Future work should incorporate targeted field monitoring (e.g., permanent plots) with stratified sampling across ecosystem types and VI trend categories, prioritizing locations near identified threshold ranges, and measuring vegetation structure/cover/biomass, soil moisture, and disturbance indicators to strengthen ecological interpretations and support management-oriented applications.

## 5. Conclusions

This study examined the temporal patterns and driving mechanisms of terrestrial ecosystem vulnerability in northwestern Sichuan. Using multi-source data, we developed a machine-learning framework (XGBoost–SHAP) that overcomes the limitations of traditional linear analyses. Using this framework, we systematically quantified the nonlinear and threshold effects of climate change and human activities on TENS vulnerability. The results showed that:

(1) The evolution of TENS vulnerability is not linear but instead shows pronounced phase transitions and spatial heterogeneity. Nearly half of the regions (47.96%) experienced nonlinear, abrupt changes in vulnerability between 1985 and 2020. The interval 2010–2015 constitutes a critical window for shifts in the state of the ecosystems. Notably, about 37.89% of the regions—primarily in eastern Ngawa Prefecture and Ganzi Prefecture—shifted from “decreasing vulnerability” to “increasing vulnerability” (D–I type). This pattern suggests that the gains from earlier ecological restoration efforts may have plateaued. Due to climate variability and accumulated human pressures, the sustainability of regional ecological restoration is therefore at serious risk, and the ecological security barrier may be vulnerable to secondary degradation.
(2) TENS vulnerability is driven by the coupled effects of multiple factors, notably human disturbance, soil moisture, and atmospheric water demand. Each driver shapes vulnerability through a distinct nonlinear threshold. GI, SM, and VPD collectively dominate the interannual variability of TENS vulnerability. This study quantified the critical ecological thresholds for these drivers. GI values below 0.90 SU/ha produced a “moderate disturbance” effect, lowering vulnerability. Beyond this threshold, GI becomes a stressor. SM exhibited a clear inflection point at 79 mm, indicating that water deficit is the primary bottleneck to ecological stability in this region. In addition, this study challenges the traditional understanding that VPD acts solely as a stress factor in arid zones. We found that high VPD within a specific range (<0.39 kPa) enhanced ecosystem resilience. This occurs because high VPD within this range indicates favorable hydrothermal coordination conditions.
(3) Different ecosystem types show distinct mechanisms driving vulnerability. These mechanisms depend strongly on habitat conditions and differ in their strategic patterns. Grassland ecosystems are constrained by the shallow soil water supply and grazing pressure. They are therefore extremely sensitive to water–human interactions. Forest and shrubland ecosystems depend more on the balance between atmospheric water demand and thermal conditions, with complex responses to climatic warming and drying. Narrow water threshold ranges govern cropland ecosystems. Wetland ecosystems are highly sensitive to human activities; even minor anthropogenic disturbance can trigger a sharp increase in vulnerability.

This study shows that current ecological management strategies need to shift from simply expanding vegetation quantity to improving ecological quality and controlling risk thresholds. In future conservation efforts, separate adaptive management strategies should be implemented for different ecosystem types. Grazing intensity in grasslands and human disturbance around wetlands should be strictly controlled. Early warning systems based on key climatic and ecological thresholds should be established to address the risk of potential regime shifts in ecosystems under climate change.

## Figures and Tables

**Table 1:** Detailed information and sources of the data used in this study.

	Name (Abbreviation)	Unit	Spatial Resolution	Temporal Resolution	Temporal Scale
1	NDVI	—	8 km	Half-month	1983–2022
2	CLCD	—	30 m	Annul	1985, 1990–2022
3	TEM	°C	1 km	Monthly	1983–2022
4	TMX	°C	1 km	Monthly	1983–2022
5	TMN	°C	1 km	Monthly	1983–2022
6	PRE	mm	1 km	Monthly	1983–2022
7	SR	kWh/m^2^	1 km	Monthly	1983–2022
8	PET	mm	4 km	Monthly	1983–2022
9	AET	mm	4 km	Monthly	1983–2022
10	SM	mm	4 km	Monthly	1983–2022
11	VPD	kPa	4 km	Monthly	1983–2022
12	RHU	%	1 km	Annual	1983–2022
13	AI	—	1 km	Annual	1983–2022
14	GI	SU/ha	1980–2000: 0.1°; 2001–2024: 0.0025°;	Annual	1983–2022
15	NTL	DN (Digital Number, dimensionless)	1 km	Annual	1984–2020

## Data Availability

The data generated in this study are publicly available on Dryad, accessible at the following link: http://datadryad.org/share/G0WwN9JNk8lbjWFUZeC95LXxXCHWtvzsfDco2njd3CQ (accessed on 15 January 2025). Other publicly archived datasets used in this study are available at the following links: https://zenodo.org/records/8253971 (accessed on 15 January 2025); https://zenodo.org/records/12779975 (accessed on 15 January 2025); https://www.geodata.cn/ (accessed on 15 January 2025); https://www.gisrs.cn/ (accessed on 15 January 2025); https://www.climatologylab.org (accessed on 15 January 2025); https://www.ncdc.ac.cn (accessed on 15 January 2025); https://data.tpdc.ac.cn/ (accessed on 15 January 2025).

## Supplementary Materials

The following supporting information can be downloaded at: https://www.mdpi.com/article/10.3390/biology15040303/s1, Figure S1: Spatial pattern of the multi-year mean Vulnerability Index (VI) during the period 1985–2020; Table S1: Proportions of area for each VI change-trend type within the TENS and within each ecosystem, expressed relative to the total TENS area for 1985–2020; Table S2: Evaluation indicators of the XGBoost model for the interannual variation in VI and its influencing factors for the entire TENS and different ecosystem types; Table S3: Variable importance rankings (%) of the factors influencing the interannual variation in VI for the entire TENS and various ecosystems, as derived from the XGBoost model. Refs. [76,77,78,79,80,81] are cited in Supplementary Materials.

## Abbreviations

The following abbreviations are used in this manuscript:

TENS	Terrestrial Ecosystem of Northwestern Sichuan
VI	Ecological Vulnerability Index
EI	Exposure Index
SI	Sensitivity Index
RI	Resilience Index
NDVI	Normalized Difference Vegetation Index
CV	Coefficient of Variation
PRE	Total precipitation
TEM	Mean temperature
TMN	Minimum temperature
TMX	Maximum temperature
SR	Total solar radiation
PET	Potential evapotranspiration
AET	Actual evapotranspiration
VPD	Vapor pressure deficit
SM	Soil moisture
AI	Aridity index
RHU	Relative humidity
GI	Grazing intensity
NTL	Artificial nighttime light
AIC	Akaike Information Criterion
VIF	Variance inflation factor
XGBoost	eXtreme Gradient Boosting
SHAP	Shapley Additive exPlanation
PDP	Partial Dependence Plot
GBDT	Gradient Boosting Decision Trees
MSE	Mean squared error
RMSE	Root mean squared error
MAE	Mean absolute error

## References

[1] Zhu Z., Piao S., Myneni R.B., Huang M., Zeng Z., Canadell J.G., Ciais P., Sitch S., Friedlingstein P., Arneth A. (2016). Greening of the Earth and its drivers. Nat. Clim. Change.

[2] Nolan C., Overpeck J.T., Allen J.R.M., Anderson P.M., Betancourt J.L., Binney H.A., Brewer S., Bush M.B., Chase B.M., Cheddadi R. (2018). Past and future global transformation of terrestrial ecosystems under climate change. Science.

[3] Xu K., Wang X.P., Jiang C., Sun O.J.X. (2020). Assessing the vulnerability of ecosystems to climate change based on climate exposure, vegetation stability and productivity. For. Ecosyst..

[4] Gonzalez P., Neilson R.P., Lenihan J.M., Drapek R.J. (2010). Global patterns in the vulnerability of ecosystems to vegetation shifts due to climate change. Glob. Ecol. Biogeogr..

[5] Yao T., Wu C., Yeh P.J.F., Hu B.X., Jiao Y. (2025). Assessing the response lag and vulnerability of terrestrial vegetation to various compound climate events in mainland China under different vegetation types. Stoch. Environ. Res. Risk Assess..

[6] Kamran M., Yamamoto K. (2023). Evolution and use of remote sensing in ecological vulnerability assessment: A review. Ecol. Indic..

[7] Fang W., Huang S.Z., Huang Q., Huang G.H., Wang H., Leng G.Y., Wang L., Guo Y. (2019). Probabilistic assessment of remote sensing-based terrestrial vegetation vulnerability to drought stress of the Loess Plateau in China. Remote Sens. Environ..

[8] Zhang Q., Yuan R.Y., Singh V.P., Xu C.Y., Fan K.K., Shen Z.X., Wang G., Zhao J.Q. (2022). Dynamic vulnerability of ecological systems to climate changes across the Qinghai-Tibet Plateau, China. Ecol. Indic..

[9] Zhang Q., Wang G., Yuan R., Singh V.P., Wu W., Wang D. (2022). Dynamic responses of ecological vulnerability to land cover shifts over the Yellow river Basin, China. Ecol. Indic..

[10] Chen Y.Y., Duo L.H., Zhao D.X., Zeng Y., Guo X.F. (2023). The response of ecosystem vulnerability to climate change and human activities in the Poyang lake city group, China. Environ. Res..

[11] Li M., Zhang X., He Y., Niu B., Wu J. (2020). Assessment of the vulnerability of alpine grasslands on the Qinghai-Tibetan Plateau. PeerJ.

[12] Weng C., Bai Y., Chen B., Hu Y., Shu J., Chen Q., Wang P. (2023). Assessing the vulnerability to climate change of a semi-arid pastoral social–ecological system: A case study in Hulunbuir, China. Ecol. Inf..

[13] Yin M.J., Yin Y.H., Zong X.Z., Deng H.Y. (2025). Global vegetation vulnerability to drought is underestimated due to the lagged effect. Agric. For. Meteorol..

[14] Jiao C.C., Yi X.B., Luo J., Wang Y., Deng Y.J., Gou J.T., Luo D.T. (2025). Spatiotemporal Dynamics of Ecological Vulnerability to Climate Change in Northwestern Sichuan’s Terrestrial Ecosystems of China: Conservation Implications. Biology.

[15] Li M., Sen C., Zhu Z., Wang Z., Myneni R., Piao S. (2023). Spatiotemporally consistent global dataset of the GIMMS Normalized Difference Vegetation Index (PKU GIMMS NDVI) from 1982 to 2022. Earth Syst. Sci. Data.

[16] Yang J., Huang X. (2024). The 30 m annual land cover datasets and its dynamics in China from 1985 to 2023. Earth Syst. Sci. Data.

[17] Peng S., Ding Y., Liu W., Li Z. (2019). 1 km monthly temperature and precipitation dataset for China from 1901 to 2017. Earth Syst. Sci. Data.

[18] Abatzoglou J.T., Dobrowski S.Z., Parks S.A., Hegewisch K.C. (2018). TerraClimate, a high-resolution global dataset of monthly climate and climatic water balance from 1958–2015. Sci. Data.

[19] Zhang L., Li X., Zheng D., Zhang K., Ma Q., Zhao Y., Ge Y. (2021). Merging multiple satellite-based precipitation products and gauge observations using a novel double machine learning approach. J. Hydrol..

[20] Hu Y., Zhang L. (2024). Added value of merging techniques in precipitation estimates relative to gauge-interpolation algorithms of varying complexity. J. Hydrol..

[21] Zhang L., Hu Y., Zhao Y., Che T. (2025). A High-Resolution Multi-Element Meteorological Driving Dataset for China via Multi-Source Data Fusion. National Cryosphere Desert Data Center (http://www.ncdc.ac.cn). https://cstr.cn/CSTR:11738.11.NCDC.NIEER.DB6722.2025.

[22] Peng S., Ding Y., Wen Z., Chen Y., Cao Y., Ren J. (2017). Spatiotemporal change and trend analysis of potential evapotranspiration over the Loess Plateau of China during 2011–2100. Agric. For. Meteorol.

[23] Ding Y., Peng S. (2020). Spatiotemporal Trends and Attribution of Drought across China from 1901–2100. Sustainability.

[24] Ding Y., Peng S. (2021). Spatiotemporal change and attribution of potential evapotranspiration over China from 1901 to 2100. Theor. Appl. Clim..

[25] Wang D., Peng Q., Li X., Zhang W., Xia X., Qin Z., Ren P., Liang S., Yuan W. (2024). A long-term high-resolution dataset of grasslands grazing intensity in China. Sci. Data.

[26] Zhang L., Ren Z., Chen B., Gong P., Fu H., Xu B. (2021). A Prolonged Artificial Nighttime-Light Dataset of China (1984–2020). National Tibetan Plateau/Third Pole Environment Data Center. http://data.tpdc.ac.cn/.

[27] Zhang L., Ren Z., Chen B., Gong P., Xu B., Fu H. (2024). A Prolonged Artificial Nighttime-light Dataset of China (1984–2020). Sci. Data.

[28] Jiao W., Wang L., Smith W.K., Chang Q., Wang H., D’Odorico P. (2021). Observed increasing water constraint on vegetation growth over the last three decades. Nat. Commun..

[29] He B., Huang L., Chen Z., Wang H. (2018). Weakening sensitivity of global vegetation to long-term droughts. Sci. China Earth Sci..

[30] Yao Y., Liu Y., Fu F., Song J., Wang Y., Han Y., Wu T., Fu B. (2024). Declined terrestrial ecosystem resilience. Glob. Change Biol..

[31] The Intergovernmental Panel on Climate Change (IPCC) (2021). Climate Change 2021: The Physical Science Basis.

[32] The Intergovernmental Panel on Climate Change (IPCC) (2022). Climate Change 2022: Impacts, Adaptation and Vulnerability.

[33] Loarie S.R., Duffy P.B., Hamilton H., Asner G.P., Field C.B., Ackerly D.D. (2009). The velocity of climate change. Nature.

[34] Seddon A.W.R., Macias-Fauria M., Long P.R., Benz D., Willis K.J. (2016). Sensitivity of global terrestrial ecosystems to climate variability. Nature.

[35] Zhang X., Zheng Y., Yang Y., Ren H., Liu J. (2025). Spatiotemporal evolution of ecological vulnerability on the Loess Plateau. Ecol. Indic..

[36] Turner B.L., Kasperson R.E., Matson P.A., McCarthy J.J., Corell R.W., Christensen L., Eckley N., Kasperson J.X., Luers A., Martello M.L. (2003). A framework for vulnerability analysis in sustainability science. Proc. Natl. Acad. Sci. USA.

[37] Li D., Wu S., Liu L., Zhang Y., Li S. (2018). Vulnerability of the global terrestrial ecosystems to climate change. Glob. Change Biol..

[38] Zhu H.L., Liu H.Z., Zhou Q.M., Cui A.H. (2023). A XGBoost-Based Downscaling-Calibration Scheme for Extreme Precipitation Events. IEEE Trans. Geosci. Remote Sens..

[39] Suganthan P.N., Kong L.P., Snásel V., Ojha V., Aly H. (2025). Euclidean and Poincaré space ensemble Xgboost. Inf. Fusion..

[40] Lundberg S.M., Lee S.I. A unified approach to interpreting model predictions. Proceedings of the 31st International Conference on Neural Information Processing Systems.

[41] R Core Team (2024). R: A Language and Environment for Statistical Computing.

[42] Esri (2020). ArcGIS Desktop.

[43] Cai X., Li Z., Liang Y. (2021). Tempo-spatial changes of ecological vulnerability in the arid area based on ordered weighted average model. Ecol. Indic..

[44] Lu Q., Zhang Y., Sun W., Wei J., Xu K. (2025). From Mountains to Basins: Asymmetric Ecosystem Vulnerability and Adaptation to Extreme Climate Events in Southwestern China. Remote Sens..

[45] Xu B., Li J., Luo Z., Wu J., Liu Y., Yang H., Pei X. (2022). Analyzing the Spatiotemporal Vegetation Dynamics and Their Responses to Climate Change along the Ya’an-Linzhi Section of the Sichuan-Tibet Railway. Remote Sens..

[46] Qu S., Wang L., Lin A., Zhu H., Yuan M. (2018). What drives the vegetation restoration in Yangtze River basin, China: Climate change or anthropogenic factors?. Ecol. Indic..

[47] Hua T., Zhao W.W., Cherubini F., Hu X.P., Pereira P. (2021). Sensitivity and future exposure of ecosystem services to climate change on the Tibetan Plateau of China. Landsc. Ecol..

[48] Yan W., Chen H., Wang S., Li Z., Wang Y., Li S., Sun R. (2021). The construction and control policy of the ecological spatial pattern of the provincial sub-regions: A case study of Northwestern Sichuan. Shanghai Urban Plan. Rev..

[49] Feng X., Fu B., Piao S., Wang S., Ciais P., Zeng Z., Lü Y., Zeng Y., Li Y., Jiang X. (2016). Revegetation in China’s Loess Plateau is approaching sustainable water resource limits. Nat. Clim. Change.

[50] Meng J., Fang H., Scavia D. (2021). Application of ecosystem stability and regime shift theories in ecosystem assessment-calculation variable and practical performance. Ecol. Indic..

[51] Hu T., Peng W., Ren Y., Zhang P. (2025). Regime shifts of vegetation ecosystems driven by human activities and climate change: A review. Acta Ecol. Sin..

[52] Kéfi S., Holmgren M., Scheffer M. (2016). When can positive interactions cause alternative stable states in ecosystems?. Funct. Ecol..

[53] Huang M.T., Zhai P.M. (2024). Impact of extreme seasonal drought on ecosystem carbon-water coupling across China. Adv. Clim. Change Res..

[54] Gartzia M., Pérez-Cabello F., Bueno C.G., Alados C.L. (2016). Physiognomic and physiologic changes in mountain grasslands in response to environmental and anthropogenic factors. Appl. Geogr..

[55] Hao Y.Q., He Z.W. (2019). Effects of grazing patterns on grassland biomass and soil environments in China: A meta-analysis. PLoS ONE.

[56] Moi D.A., García-Ríos R., Hong Z., Daquila B.V., Mormul R.P. (2020). Intermediate disturbance hypothesis in ecology: A literature review. Ann. Zool. Fenn..

[57] Sun S.N., Zhao Y., Dong Q.M., Yang X.X., Liu Y.Z., Liu W.T., Shi G., Liu W.T., Zhang C.P., Yu Y. (2023). Symbiotic diazotrophs in response to yak grazing and Tibetan sheep grazing in Qinghai-Tibetan plateau grassland soils. Front. Microbiol..

[58] Zhu Q.A., Chen H., Peng C.H., Liu J.X., Piao S.L., He J.S., Wang S.P., Zhao X.Q., Zhang J., Fang X.Q. (2023). An early warning signal for grassland degradation on the Qinghai-Tibetan Plateau. Nat. Commun..

[59] Guo Q., Li S., Hu Z., Zhao W., Yu G., Sun X., Li L., Liang N., Bai W. (2016). Responses of gross primary productivity to different sizes of precipitation events in a temperate grassland ecosystem in Inner Mongolia, China. J. Arid. Land..

[60] Fu Z., Ciais P., Wigneron J.-P., Gentine P., Feldman A.F., Makowski D., Viovy N., Kemanian A.R., Goll D.S., Stoy P.C. (2024). Global critical soil moisture thresholds of plant water stress. Nat. Commun..

[61] Jurado S., Matthes J. (2025). Increasing Large Precipitation Events and Low Available Water Holding Capacity Create the Conditions for Dry Land-Atmosphere Feedbacks in the Northeastern United States. Water Resour. Res..

[62] Novick K.A., Ficklin D.L., Stoy P.C., Williams C.A., Bohrer G., Oishi A.C., Papuga S.A., Blanken P.D., Noormets A., Sulman B.N. (2016). The increasing importance of atmospheric demand for ecosystem water and carbon fluxes. Nat. Clim. Change.

[63] Yuan W., Zheng Y., Piao S., Ciais P., Lombardozzi D., Wang Y., Ryu Y., Chen G., Dong W., Hu Z. (2019). Increased atmospheric vapor pressure deficit reduces global vegetation growth. Sci. Adv..

[64] Grossiord C., Buckley T.N., Cernusak L.A., Novick K.A., Poulter B., Siegwolf R.T.W., Sperry J.S., McDowell N.G. (2020). Plant responses to rising vapor pressure deficit. New Phytol..

[65] Tian Y., Zhang W., Xu X., Zhou B., Cao X., Qiao B. (2025). Response of Water-Use Efficiency (WUE) in Alpine Grasslands to Hydrothermal and Radiative Factors Across Elevation Gradients. Land.

[66] Xing Y., Chen M., Dao J., Lin L., Chen C., Chen Y., Wang Z. (2024). Fine-root morphology of woody and herbaceous plants responds differently to altered precipitation: A meta-analysis. For. Ecol. Manag..

[67] Xue Y., Liang H., Ma Y., Xue G., He J. (2023). The Impacts of Climate and Human Activities on Grassland Productivity Variation in China. Remote Sens..

[68] Yang G.S., Huang L., Shi Y.F. (2022). Magnitude and determinants of plant root hydraulic redistribution: A global synthesis analysis. Front. Plant Sci..

[69] Shi C., Schneider L., Hu Y., Shen M., Sun C., Xia J., Forbes B.C., Shi P., Zhang Y., Ciais P. (2020). Warming-induced unprecedented high-elevation forest growth over the monsoonal Tibetan Plateau. Environ. Res. Lett..

[70] Zhou S., Wu S., Gao J., Liu L., Li D., Yan R., Wang J. (2024). Increased stress from compound drought and heat events on vegetation. Sci. Total Environ..

[71] Feng X., Liu R., Li C., Zhang H., Slot M. (2023). Contrasting responses of two C_4_ desert shrubs to drought but consistent decoupling of photosynthesis and stomatal conductance at high temperature. Environ. Exp. Bot..

[72] Rasheed M.W., Tang J., Sarwar A., Shah S., Saddique N., Khan M.U., Imran Khan M., Nawaz S., Shamshiri R.R., Aziz M. (2022). Soil Moisture Measuring Techniques and Factors Affecting the Moisture Dynamics: A Comprehensive Review. Sustainability.

[73] Chen L., Deng X., Duan H., Tan X., Xie X., Pan X., Guo L., Luo T., Chen X., Gao H. (2025). Canopy humidity and irrigation regimes interactively affect rice physiology, grain filling and yield during grain filling period. Agric. Water Manag..

[74] Gao C., Knorr K.-H., Chen H., He Y. (2023). Editorial: Disturbance, resilience, and restoration of wetlands. Front. Ecol. Evol..

[75] Eresanya O.I., Babaniyi B.R., Ojo O.T., Olorunfemi K.O., Babaniyi B.R., Aransiola S.A., Babaniyi E.E., Maddela N.R. (2025). Future Challenges and Opportunities for Wetland Preservation. Wetland Ecosystems: Conservation Strategies, Policy Management and Applications.

[76] Tang J., Niu B., Fu G., Peng J., Hu Z., Zhang X. (2025). Shifted trend in drought sensitivity of vegetation productivity from 1982 to 2020. Agric. For. Meteorol..

[77] Ippolito A., Sala S., Faber J.H., Vighi M. (2010). Ecological vulnerability analysis: A river basin case study. Sci. Total Environ..

[78] De Keersmaecker W., Lhermitte S., Tits L., Honnay O., Somers B., Coppin P. (2015). A model quantifying global vegetation resistance and resilience to short-term climate anomalies and their relationship with vegetation cover. Glob. Ecol. Biogeogr..

[79] Simoniello T., Lanfredi M., Liberti M., Coppola R., Macchiato M. (2008). Estimation of vegetation cover resilience from satellite time series. Hydrol. Earth Syst. Sci. Discuss..

[80] Carpenter S.R., Cole J.J., Pace M.L., Batt R., Brock W.A., Cline T., Coloso J., Hodgson J.R., Kitchell J.F., Seekell D.A. (2011). Early warnings of regime shifts: A whole-ecosystem experiment. Science.

[81] Kling M.M., Auer S.L., Comer P.J., Ackerly D.D., Hamilton H. (2020). Multiple axes of ecological vulnerability to climate change. Glob. Change Biol..

