# Supplementary material for: Interannual Regime Shifts and Driver Thresholds of Terrestrial Ecosystem Vulnerability in Northwestern Sichuan of China Based on an XGBoost-SHAP Model
Source: Biology (Basel). 2026 Feb 9;15(4):303. doi: 10.3390/biology15040303 (PMC12938296; doi:10.3390/biology15040303)
Supplement: Supplementary file 1 [file biology-15-00303-s001.zip › biology-4115006-supplementary.pdf]

Interannual Regime Shifts and Driver Thresholds of Terrestrial Ecosystem Vulnerability in Northwestern Sichuan of China Based on an XGBoost-SHAP Model

Cuicui Jiao [1,*], Zonggui He [2], Juan Xu [3], Xiaobo Yi [1], Ji Luo [1] and Ping Huang [1]

[1] School of Economics, Sichuan University of Science & Engineering, Yibin 644000, China
[2] School of Economics, Guizhou University, Guiyang 550025, China; hezonggui1997@163.com
[3] College of Economics and Management, Hebei Agricultural University, Baoding 071000, China
* Correspondence: dingding20202026@163.com

Ecological vulnerability assessment

In this study, vulnerability was evaluated following the same conceptual framework and methodological approach as that adopted by Jiao et al. [1]. The detailed information is as follows.

(1) Data preprocessing

A sliding window approach was adopted to facilitate the analysis of temporal trends in vulnerability and to minimize uncertainties in the assessment model caused by extreme climatic or other sudden events. The principle of this method is to divide the entire dataset into relatively small, continuous time segments by setting a fixed unit interval (i.e., a window) and moving it step by step across the series. This approach is commonly employed in long-term change research of vegetation growth [2-4]. In this study, monthly NDVI, temperature, and precipitation data from 1983 to 2022 were analyzed using a five-year moving window length and a one-year step size, starting from 1983 and moving forward annually to end in 2022, to generate 36 overlapping periods of 1983-1987, 1984-1989, ……., 2018-2022, every period containing 60 months. The five-year window was chosen as it is long enough to buffer the effects of short-term climate oscillations (e.g., El Niño-Southern Oscillation) and anomalous years, thus capturing more stable ecosystem responses to climate trends [2, 4]. At the same time, this window size is short enough to detect decadal-scale shifts in vulnerability, providing a balance between signal stability and temporal resolution [2, 4].

(2) Overview of the ecological vulnerability

In this study, the ecological vulnerability to climate change was assessed within a widely used framework of exposure, sensitivity, and resilience. Exposure refers to the extent of climate disturbance that terrestrial ecosystems may undergo [5]. Sensitivity refers to the extent to which an ecosystem may be affected under a certain disturbance [6,7]. Resilience refers to an ecosystem's capacity to return to its original state after a disturbance [8]. The assessment model for ecological vulnerability was developed as follows (Equation S1), by incorporating these three components into an overall vulnerability index, as proposed by Li et al. [9].

$$VI = \sqrt{\frac{EI \times SI}{1+RI}} \qquad \text{(S1)}$$

where, VI is the Ecological Vulnerability Index; EI is the Exposure Index, SI is the Sensitivity Index, and RI is the Resilience Index. This model integrates the three core components of vulnerability. EI and SI represent the potential impact, while RI acts as a mitigating factor. Vulnerability increases with greater exposure and sensitivity but decreases with higher resilience. This formulation ensures that resilience alone cannot eliminate vulnerability unless the initial impact (EI or SI) is zero. The final

VI represents a synthesized measure of the ecosystem's susceptibility to harm from climate change. It thus reaches its peak when resilience is absent, given the functions of EI and SI [10]. Its effectiveness in identifying vulnerable areas has been demonstrated in previous studies [9,11,12].

(3) Resilience, Sensitivity and Exposure

Vegetation dynamics show long-term memory, being influenced by current climate and past states, with ecosystems exhibiting persistence [13]. Accordingly, resilience can be quantified via autoregressive (AR) coefficients associated with vegetation restoration time and persistence [14], derived from prior fitting [15]. We applied an AR (1) multiple linear regression within each five-year sliding window (60 months) to characterize ecosystem responses to climate change at the grid scale (Equation S2):

$$NDVI_t = \alpha \times Temp_t + \beta \times Pre_t + \gamma \times NDVI_{t-1} + \varepsilon_t \qquad \text{(S2)}$$

where $NDVI_t$ and $NDVI_{t-1}$ are the monthly standardized NDVI anomalies at time t and $t-1$. $Temp_t$ and Pre_t are the monthly standardized temperature and precipitation anomalies at time t. The parameters α, β, and γ are the fitting coefficients, and ε_t is the residual term. To ensure comparability between model coefficients, monthly NDVI, temperature, and precipitation data within each five-year sliding window were standardized using the z-score method [7]. The coefficients α, β, and γ were normalized to a 0–1 range using min-max scaling that ensures scale-invariant indices without bias from variable magnitudes.

The coefficients in the AR (1) model (Equation S2) are intrinsically linked to the three dimensions of ecological vulnerability. The parameter γ captures the degree of dependence of current vegetation on its immediate past state, and thus reflects the ecosystem's memory effect. Consequently, the Resilience Index (RI) can be defined as follows (Equation S3) [13]:

$$RI = 1 - \gamma \qquad \text{(S3)}$$

where RI represents the Resilience Index, and γ is the approximate value of $NDVI_{t-1}$ in Equation (S2).

The parameters α and β quantify the magnitude of vegetation response to instantaneous changes in temperature and precipitation. Higher absolute values indicate lower resistance to climate fluctuations. Thus, the Exposure Index (EI) can be defined as follows (Equation S4) [9,16]:

$$EI = \alpha + \beta \qquad \text{(S4)}$$

where EI is the Exposure Index; α and β are the fitting coefficients of temperature and precipitation anomalies in Equation S2.

Sensitivity Index (SI) can be obtained by the weighted summation of the standardized meteorological anomalies and related fitting coefficients [7], as follows (Equation S5):

$$SI = \alpha \times T_{-norm} + \beta \times P_{-norm} \qquad \text{(S5)}$$

where SI is the Sensitivity Index, and α and β are the fitting coefficients of temperature and precipitation anomalies in Equation S2. T_{-norm} and P_{-norm} represent the mean normalized temperature and precipitation within each five-year window, respectively.

Based on a 1 km × 1 km grid cell scale, the EI, SI, RI, and VI were calculated using Equations (S1) – (S5) for each sliding window (1983–1987, 1984–1988, ..., 2018–2022). The midpoint year of each window (1985, 1986, ..., 2020) was used as the temporal reference, yielding in continuous time series data for each index from 1985 to 2020.

Figure S1 Spatial pattern of the multi-year mean Vulnerability Index (VI) during the period 1985–2020.

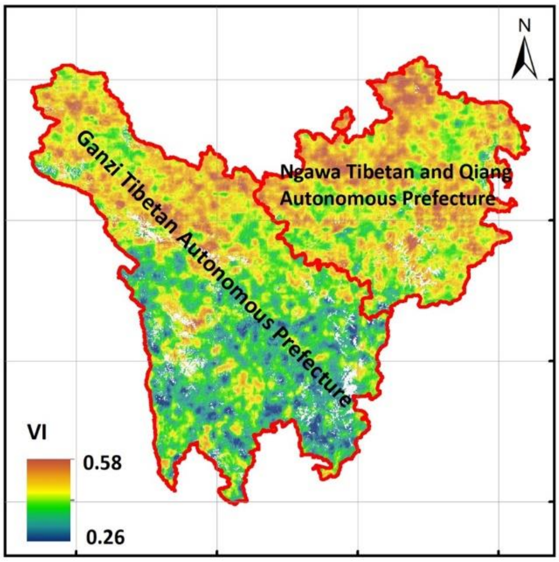

Table S1 Proportions of area for each VI change-trend type within the TENS and within each ecosystem, expressed relative to the total TENS area for 1985–2020.

<table>
<tr><th></th><th colspan="3">CV (%)</th><th colspan="4">Types of trends</th><th colspan="2">Reversal types</th><th colspan="5">Turning point (year)</th></tr>
<tr><th></th><th><20%</th><th>20%-30%</th><th>>30%</th><th>No trend</th><th colspan="2">Linear</th><th>Abrupt</th><th>I–D</th><th>D–I</th><th>1990–1995</th><th>1995–2000</th><th>2000–2005</th><th>2005–2010</th><th>2010–2015</th></tr>
<tr><td rowspan="3">TENS</td><td rowspan="3">31.53</td><td rowspan="3">59.36</td><td rowspan="3">9.11</td><td rowspan="3">40.42</td><td>positive</td><td>negative</td><td rowspan="3">47.96</td><td rowspan="3">9.89</td><td rowspan="3">38.07</td><td rowspan="3">7.68</td><td rowspan="3">9.93</td><td rowspan="3">10.12</td><td rowspan="3">6.32</td><td rowspan="3">13.92</td></tr>
<tr><td>6.96</td><td>4.66</td></tr>
<tr><td colspan="2">11.62</td></tr>
<tr><td>Cropland</td><td>0.06</td><td>0.17</td><td>0.01</td><td>0.12</td><td colspan="2">0.02</td><td>0.11</td><td>0.01</td><td>0.10</td><td>0.02</td><td>0.03</td><td>0.02</td><td>0.02</td><td>0.03</td></tr>
<tr><td>Forest</td><td>6.09</td><td>18.21</td><td>3.89</td><td>11.22</td><td colspan="2">3.06</td><td>13.91</td><td>2.75</td><td>11.15</td><td>2.29</td><td>3.03</td><td>2.69</td><td>1.89</td><td>4.02</td></tr>
<tr><td>Shrubland</td><td>0.54</td><td>1.07</td><td>0.18</td><td>0.71</td><td colspan="2">0.22</td><td>0.87</td><td>0.18</td><td>0.69</td><td>0.13</td><td>0.17</td><td>0.18</td><td>0.12</td><td>0.26</td></tr>
<tr><td>Grassland</td><td>24.52</td><td>39.77</td><td>5.02</td><td>28.05</td><td colspan="2">8.28</td><td>32.98</td><td>6.89</td><td>26.08</td><td>5.20</td><td>6.69</td><td>7.21</td><td>4.27</td><td>9.60</td></tr>
<tr><td>Wetland</td><td>0.33</td><td>0.14</td><td>0.00</td><td>0.32</td><td colspan="2">0.04</td><td>0.10</td><td>0.05</td><td>0.05</td><td>0.04</td><td>0.02</td><td>0.01</td><td>0.03</td><td>0.01</td></tr>
</table>

Table S2 Evaluation indicators of the XGBoost model between the interannual variation of VI and its influencing factors for the entire TENS and different ecosystem types

	R^2	RMSE	MSE	MAE
TENS	0.937	0.010	0.000	0.006
Cropland	0.983	0.006	0.000	0.004
Forest	0.998	0.002	0.000	0.001
Shrubland	0.989	0.004	0.000	0.003
Grassland	0.711	0.021	0.000	0.016
Wetland	0.600	0.039	0.001	0.027

Table S3 Variable importance rankings (%) of the factors influencing the VI interannual variation in VI for the entire TENS and various ecosystems, as derived from the XGBoost model.

TENS	Feature	GI	SM	VPD	PRE	TMX	TMN	SR	AET	
	Importance (Gain)	21.59%	19.03%	18.35%	10.24%	8.64%	7.63%	7.40%	7.13%	
Cropland	Feature	RHU	SM	SR	TMX	AI	GI			
	Importance (Gain)	25.11%	21.05%	16.95%	15.31%	13.25%	8.34%			
Forest	Feature	VPD	TMN	PRE	AET	TMX	NTL	SR	TEM	
	Importance (Gain)	33.28%	17.02%	11.60%	10.14%	9.94%	7.42%	5.71%	4.88%	
Shrubland	Feature	VPD	SM	TMX	PRE	TMN	TEM	NTL	SR	
	Importance (Gain)	23.05%	17.70%	15.77%	13.96%	13.37%	6.28%	6.27%	3.60%	
Grassland	Feature	SM	GI	TMX	TMN	AET	VPD	SR	PRE	TEM
	Importance (Gain)	24.54%	16.38%	11.47%	11.05%	9.12%	8.12%	7.83%	6.86%	4.64%
Wetlands	Feature	VPD	NTL	SR	SM	GI	AI	TMX	TMN	
	Importance (Gain)	18.45%	14.49%	13.96%	13.53%	11.56%	9.79%	9.26%	8.94%	

References

1. Jiao, C.C.; Yi, X.B.; Luo, J.; Wang, Y.; Deng, Y.J.; Gou, J.T.; Luo, D.T. Spatiotemporal Dynamics of Ecological Vulnerability to Climate Change in Northwestern Sichuan's Terrestrial Ecosystems of China: Conservation Implications. *Biology-Basel* **2025**, *14*, 1625.
2. Jiao, W.; Wang, L.; Smith, W.K.; Chang, Q.; Wang, H.; D'Odorico, P. Observed increasing water constraint on vegetation growth over the last three decades. *Nat. Commun.* **2021**, *12*, 3777.
3. Tang, J.; Niu, B.; Fu, G.; Peng, J.; Hu, Z.; Zhang, X. Shifted trend in drought sensitivity of vegetation productivity from 1982 to 2020. *Agric. For. Meteorol.* **2025**, *362*, 110388.
4. Yao, Y.; Liu, Y.; Fu, F.; Song, J.; Wang, Y.; Han, Y.; Wu, T.; Fu, B. Declined terrestrial ecosystem resilience. *Glob Chang Biol.* **2024**, *30*, e17291.
5. Loarie, S.R.; Duffy, P.B.; Hamilton, H.; Asner, G.P.; Field, C.B.; Ackerly, D.D. The velocity of climate change. *Nature* **2009**, *462*, 1052-1055.
6. Zhang, X.; Zheng, Y.; Yang, Y.; Ren, H.; Liu, J. Spatiotemporal evolution of ecological vulnerability on the Loess Plateau. *Ecol. Indic.* **2025**, *170*, 113060.
7. Seddon, A.W.R.; Macias-Fauria, M.; Long, P.R.; Benz, D.; Willis, K.J. Sensitivity of global terrestrial ecosystems to climate variability. *Nature* **2016**, *531*, 229-232.
8. Turner, B.L.; Kasperson, R.E.; Matson, P.A.; McCarthy, J.J.; Corell, R.W.; Christensen, L.; Eckley, N.; Kasperson, J.X.; Luers, A.; Martello, M.L.; et al. A framework for vulnerability analysis in sustainability science. *Proc. Natl. Acad. Sci. U.S.A.* **2003**, *100*, 8074-8079.
9. Li, D.; Wu, S.; Liu, L.; Zhang, Y.; Li, S. Vulnerability of the global terrestrial ecosystems to climate change. *Global Change Biol.* **2018**, *24*, 4095-4106.
10. Ippolito, A.; Sala, S.; Faber, J.H.; Vighi, M. Ecological vulnerability analysis: A river basin case study. *Sci. Total Environ.* **2010**, *408*, 3880-3890.
11. Zhang, Q.; Wang, G.; Yuan, R.; Singh, V.P.; Wu, W.; Wang, D. Dynamic responses of ecological vulnerability to land cover shifts over the Yellow river Basin, China. *Ecol. Indic.* **2022**, *144*, 109554.
12. Zhang, Q.; Yuan, R.; Singh, V.P.; Xu, C. Y.; Fan, K.; Shen, Z.; Wang, G.; Zhao, J. Dynamic vulnerability of ecological systems to climate changes across the Qinghai-Tibet Plateau, China. *Ecol. Indic.* **2022**, *134*, 108483.
13. De Keersmaecker, W.; Lhermitte, S.; Tits, L.; Honnay, O.; Somers, B.; Coppin, P. A model quantifying global vegetation resistance and resilience to short-term climate anomalies and their relationship with vegetation cover. *Global Ecol. Biogeogr.* **2015**, *24*, 539-548.
14. Simoniello, T.; Lanfredi, M.; Liberti, M.; Coppola, R.; Macchiato, M. Estimation of vegetation cover resilience from satellite time series. *Hydrol. Earth Syst. Sci. Discuss.* **2008**, *12*, 1053–1064.
15. Carpenter, S.R.; Cole, J.J.; Pace, M.L.; Batt, R.; Brock, W.A.; Cline, T.; Coloso, J.; Hodgson, J.R.; Kitchell, J.F.; Seekell, D.A.; et al. Early warnings of regime shifts: a whole-ecosystem experiment. *Science* **2011**, *332*, 1079-1082.
16. Kling, M.M.; Auer, S.L.; Comer, P.J.; Ackerly, D.D.; Hamilton, H. Multiple axes of ecological vulnerability to climate change. *Global Change Biol.* **2020**, *26*, 2798-2813.
